# Flexible Neuromorphic Memristors: From Mechanisms to Applications

**DOI:** 10.3390/ma19153234

**Published:** 2026-07-30

**Authors:** Letian Yang, Jing Cheng, Yun Zhang, Yunbo Wang, Jiseng Yao, Yuqing Liu

**Affiliations:** College of Textile and Clothing Engineering, Soochow University, Suzhou 215123, China

**Keywords:** flexible memristor, neuromorphic computing, resistive switching mechanism, synaptic plasticity, in-memory computing

## Abstract

The von Neumann architecture, due to the physical separation between memory and processor, has limited the development of data-intensive applications. Neuromorphic computing technologies inspired by the brain’s parallel and event-driven operation mechanisms have enabled low-power in-memory computing. Memristors with tunable conductance can emulate biological synapses, while flexible memristors further offer mechanical flexibility, making them suitable for wearable electronics and intelligent sensing systems. This review systematically summarizes the switching mechanisms of flexible neuromorphic memristors, including conductive filaments, interface effects, ferroelectricity, phase change, and multiple synergistic mechanisms. It categorically discusses natural and bio-derived materials, synthetic organic/polymer materials, and inorganic functional materials, and introduces strategies for enhancing flexibility. The article also covers device architectures such as sandwich structures, crossbar arrays, and fiber-based textile structures, along with low-temperature fabrication techniques. Finally, it reviews recent advances in neuromorphic computing, in-memory computing, biomimetic sensing, and biomedical wearable systems. Challenges related to mechanical stability and device uniformity are analyzed, and future directions toward self-healing materials and integrated sensing-storage-computing systems are outlined. This comprehensive review bridges the gap between material innovation and system-level integration in flexible neuromorphic memristors, providing a valuable roadmap for accelerating the development of next-generation wearable artificial intelligence, edge computing, and bio-integrated electronic technologies.

## 1. Introduction

The conventional von Neumann computing architecture is constrained by the physical separation of memory and processing, leading to severe data transfer latency and energy dissipation, which makes it difficult to meet the demands of data-intensive applications such as big data and artificial intelligence [1,2,3]. Neuromorphic computing, inspired by the highly parallel, event-driven operating mechanism of neurons and synapses in the human brain [4], achieves in-memory computing, low power consumption, and high fault tolerance, and is regarded as one of the most promising technological routes in the post-Moore era [5]. The memristor, as a core component of neuromorphic computing, leverages its two-terminal structure and the essential feature that its resistance state can be modulated by the voltage history to efficiently emulate various forms of synaptic plasticity [6,7], thereby providing an ideal hardware foundation for constructing efficient neuromorphic computing systems.

Unlike conventional memory cells, the continuously tunable conductance of memristors makes them naturally suitable for synaptic weight updates, demonstrating unique advantages in in-memory computing architectures [8]. Over the past few years, memristors based on various material systems have successfully emulated key synaptic functions, including spike-timing-dependent plasticity (STDP) and long-term potentiation/depression (LTP/LTD) [9,10]. Meng et al. [7] fabricated memristors using hexagonal boron nitride (h-BN) nanosheets. By electrically modulating the gradual evolution of boron-vacancy conductive filaments, they successfully emulated both asymmetric and symmetric STDP learning rules, achieving an ultralow energy consumption of 140 fJ per synaptic event and a response speed of 1 μs. Wang et al. [8] fabricated ZnO-based flexible memristors via physical vapor deposition at room temperature, achieving highly linear LTP/LTD conductance modulation. Furthermore, the conductance modulation of memristors can also be volatile. Volatile memristors based on organic materials, two-dimensional anisotropic crystals, and oxide heterojunctions exhibit spontaneous decay of their resistance states upon removal of the stimulus, which naturally lends itself to emulating the short-term forgetting function of biological synapses. Wang et al. [11] leveraged the in-plane anisotropy of layered NbOCl_2_ to achieve forming-free volatile resistive switching through directional oxygen ion migration, successfully emulating short-term plasticity behaviors such as excitatory postsynaptic current (EPSC). Li et al. [12] constructed a Pt/TiO_x_/NiO_x_/Au memristor on a coaxial optical fiber, which exhibited volatile integrate-and-fire neuron characteristics under electrical signals while mimicking nonvolatile synaptic behavior under optical signals, with stable performance at bending radii as small as 4 mm. These milestones mark the evolution of memristors from simple nonvolatile memory elements into core hardware building blocks that support efficient neuromorphic computing. With the rapid development of emerging fields such as wearable electronic devices, electronic skin, and implantable medical systems, modern society urgently demands electronic components that can seamlessly integrate with the human body, adapt to complex curved surfaces, and withstand repeated mechanical deformation [13,14]. However, although conventional rigid memristors exhibit excellent electrical characteristics and neuromorphic computing potential, their brittle nature and incompatibility with flexible interfaces severely hinder their application in body-attached intelligent systems [15]. Against this backdrop, flexible memristors have emerged. They not only retain the core advantages of memristors—nonvolatility, low power consumption, and analog synaptic plasticity—but also achieve excellent mechanical flexibility through substrate engineering, material selection, and structural innovation [16,17]. For example, Kim et al. [18] fabricated nitrogen-doped tantalum oxide (N:TaO_x_) memristor crossbar arrays on flexible polyethylene terephthalate (PET) substrates using low-temperature atomic layer deposition at 150 °C. After 10^4^ bending cycles with a bending radius of 2.5 mm, the switching performance showed no significant degradation, and uniform resistive switching characteristics with pattern storage were achieved in a 6 × 6 array. Yuklyaevskikh et al. [19] systematically investigated the electrical behavior of parylene-based flexible memristors under mechanical bending and found that the devices operated stably even at a bending radius as small as 0.5 cm, and the current-voltage (I–V) characteristics remained largely unchanged after 100 bend-flatten cycles. Currently, flexible memristors have been successfully integrated into prototype systems such as tactile sensing arrays for electronic skin and wearable health monitoring patches, demonstrating great potential to replace traditional rigid devices and construct next-generation bionic and human-integrated intelligent hardware [14,15,17]. Huang et al. [20] constructed flexible sensory neurons based on transition metal carbide/nitride (MXene), which achieved 93.21% accuracy in handwritten digit recognition, although long-term stability after repeated bending still requires validation. Lee et al. [21] developed a 32 × 32 self-rectifying synaptic array that achieved 93.5% accuracy in electrocardiogram diagnosis; however, low-temperature process compatibility and device-to-device uniformity remain application bottlenecks.

Although significant progress has been made in the research of flexible neuromorphic memristors, key challenges such as mechanical stability, device uniformity, and low-temperature process compatibility persist in practical applications [18,22]. Shooshtari et al. [23] reviewed the applications of memristors in in-memory computing and spiking neural networks, systematically elaborating on the implementation frameworks at the device–circuit–system levels. However, their work was primarily based on rigid substrates and standard CMOS processes, without addressing the unique constraints imposed by mechanical deformation on device performance and system integration. Meanwhile, systematic review studies on the material systems, switching mechanisms, structural designs, and system applications of flexible memristors remain scarce in the current literature. In light of this research status and the existing gaps, this review systematically and comprehensively summarizes the latest research progress, core technological frameworks, current bottlenecks, and future development trends of flexible neuromorphic memristors. We first summarize the physical mechanisms of resistive switching and their synergistic multi-mechanism dynamics, with particular emphasis on multi-mechanism synergy in flexible systems, in contrast to the predominant single-filament or interface-model descriptions in rigid memristors. Subsequently, focusing on functional layer materials, we categorize and discuss the flexibility adaptation strategies and synaptic performance of natural and bio-derived materials, synthetic organic/polymer materials, and inorganic functional materials and nanofillers. We systematically evaluate the retention stability of resistive switching under mechanical loads such as bending and stretching for each material system, and particularly highlight the constraints imposed by batch-to-batch variability of natural materials on device reproducibility. On this basis, it systematically introduces device structures (sandwich, crossbar arrays, and fiber/textile structures) and low-temperature fabrication techniques (atomic layer deposition, solution-based methods, and fully printing processes), and evaluates flexible substrate selection and mechanical stability testing methods. At the application level, this review summarizes recent progress in four directions: neuromorphic computing, in-memory computing, biomimetic sensing, and biomedical/wearable systems. Finally, key challenges such as mechanical stability, device uniformity, low-temperature process compatibility, and standardized testing are analyzed, and future directions including self-healing materials, three-dimensional integration, and integrated sensing-memory-computing systems are discussed. As a critical bridge connecting neuromorphic computing and flexible electronics, flexible memristors are expected to play an irreplaceable role in next-generation wearable intelligent hardware, biomimetic sensing systems, and edge computing. This review aims to provide systematic reference and inspiration for in-depth research and expanded applications in this field.

## 2. Resistive Switching Mechanisms of Flexible Memristors

**Figure 1:**
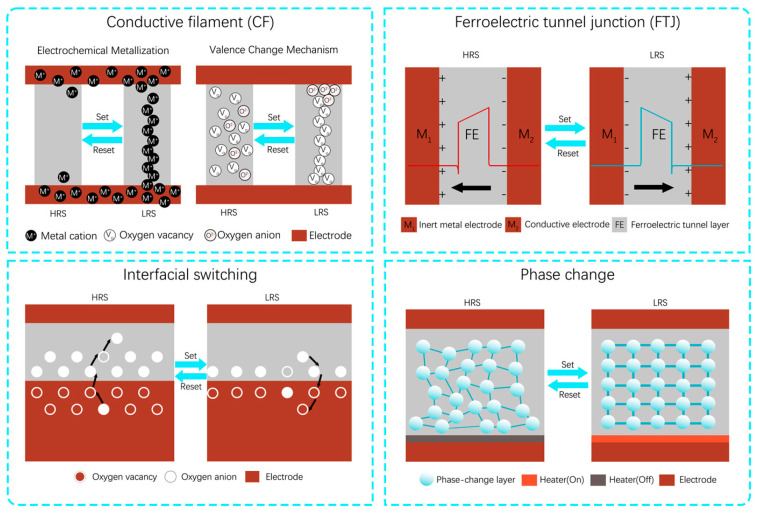
Mechanisms of flexible memristors.

The resistive switching (RS) mechanism of flexible memristors is the physical foundation for realizing neuromorphic computing functionalities. Its essence lies in the reversible switching of resistance states driven by electric-field-induced ion migration, interfacial barrier modulation, or phase transitions within the functional layer. Based on the dominant mechanisms, RS can be classified into conductive filament, interfacial, ferroelectric tunnel junction, and phase-change/Mott types. Each mechanism has its own advantages and disadvantages in terms of linearity, uniformity, power consumption, and endurance, while multi-mechanism synergy further expands functional programmability. The following sections elaborate on the fundamental principles, recent advances, and application challenges of each mechanism in flexible neuromorphic devices (Figure 1).

### 2.1. Conductive Filament Resistive Switching Mechanism

The conductive filament (CF) mechanism is the core physical basis for reversible resistance switching in memristors, essentially involving the formation or rupture of highly conductive microscopic channels within the functional layer under an external electric field. Depending on the source and evolution path of the filament material, this mechanism can be divided into electrochemical metallization (ECM) and valence change mechanism (VCM) [24,25,26]. The ECM mechanism relies on the electrochemical redox reaction of an active metal electrode (Cu, Ag, etc.): a forward bias oxidizes the metal anode to generate cations, which migrate to the counter electrode, where they are reduced, nucleate, and grow into metallic filaments; a reverse bias causes electrochemical dissolution of the filament, resetting the device to a high-resistance state (HRS). In contrast, the VCM does not depend on metal ions, but is driven by the electric-field-induced migration and redistribution of oxygen vacancies in transition metal oxides: oxygen vacancies drift and aggregate under the electric field to form conductive paths, setting the device to a low-resistance state (LRS), while a reverse bias promotes the recombination of vacancies with oxygen ions and filament rupture to achieve reset [27,28].

The difference in synaptic linearity between ECM and VCM stems from the distinct nature of filament evolution. In ECM, the reduction and nucleation of metal cations is a typical percolation phase transition process, often resulting in abrupt filament growth and thus high nonlinearity in conductance modulation. In VCM, the formation and dissolution of oxygen-vacancy filaments depend on continuous modulation of defect concentration, and the conductance change has a smoother correspondence with the amount of vacancy migration, thus offering advantages in simulating gradual synaptic weight updates [28,29,30]. Shooshtari et al. [31] achieved gradual conductance changes for long-term potentiation and depression in HfO_2_-based VCM memristors using continuous pulse sequences, with minimal variation over 100 independent STDP tests (<±2% under voltage perturbation and <±2.5% under timing jitter), fully validating the superiority of the VCM for synaptic plasticity emulation. However, noticeable degradation of the LRS after 100 cycles also exposed the limitation of VCM in long-term reliability. In terms of device reliability, the main challenge for ECM lies in the significant randomness in the nucleation position and growth path of metallic filaments. Lee et al. [32] observed a wide distribution of Reset voltages ranging from −0.1 V to −0.7 V in Cu_2-x_S-based ECM memristors, with statistical data from 50 independent devices showing that some devices exhibited an ON/OFF ratio below 10^3^, directly reflecting the insufficient device-to-device uniformity of the ECM mechanism. To address these respective shortcomings, researchers have developed three main improvement routes. First, doping and nanoparticle confinement. Pan et al. [33] doped WO_3−x_ with TiO_2_, reducing the nonlinearity factor of long-term potentiation from 0.023 to 0.008 and that of depression from −0.037 to −0.014, achieving 98.7% accuracy on the MNIST dataset (compared to 93.4% for the undoped device). Zuo et al. [29] embedded Ag nanoparticles in a-SiC_0.11_:H, leveraging localized electric field enhancement to reduce the coefficient of variation for Set and Reset voltages by 76.2% and 69.7%, respectively, with an ON/OFF ratio as high as 10^7^. Second, bilayer structure for nucleation guidance. Saleem et al. [30] utilized the concentration gradient between a TaO_x_ layer (36% oxygen vacancy concentration) and an HfO_x_ layer (29%) to guide filament nucleation at designated sites, reducing the resistance-state coefficient of variation by more than half, with stable performance maintained after 1000 cycles at a bending radius of 4 mm. Third, interfacial engineering with two-dimensional materials. Kumar et al. [34] introduced a monolayer graphene barrier layer for Cu^+^ injection in V-doped Ga_2_O_3_; control experiments showed that devices without graphene exhibited abrupt switching and large fluctuations, while those with graphene displayed gradual, self-limited switching over 100 DC cycles, with switching voltages reduced from ±4.5 V to ±2.3 V at a bending radius of 10 mm. In summary, ECM offers advantages of high ON/OFF ratio and fast speed, but its randomness limits linearity and reliability; VCM performs better in linearity and uniformity, but its long-term cycling stability needs improvement. Doping, multilayer structures, and 2D material engineering can effectively compensate for the respective shortcomings, promoting the development of conductive-filament-type memristors toward highly reliable flexible neuromorphic computing.

### 2.2. Interface-Type and Bulk Trap Modulation Mechanisms

The interface-type resistive switching mechanism differs from the conductive filament type in that the resistance change does not arise from the formation and rupture of conductive pathways inside the functional layer, but rather from the modulation of the Schottky barrier height at the interface region between the metal electrode and the functional layer by an external electric field, enabling reversible switching between high- and low-resistance states. Yang et al. [35] systematically confirmed this mechanism in Cs_3_Cu_2_I_5_-based flexible memristors: the resistance change originated from the modification of the depletion layer width at the Au/Cs_3_Cu_2_I_5_ interface caused by iodine vacancy migration, and the current scaled linearly with the electrode area (rather than showing point-contact conduction characteristic of filamentary type), thereby achieving a write resistance as high as 243 MΩ and sub-femtojoule energy consumption.

During the interfacial barrier modulation process, charge trapping and detrapping effects often play a critical role, including carrier capture by interface states as well as bulk traps modulating bulk carrier mobility through space-charge effects. Li et al. [36] inserted a pyridinium salt buffer layer between graphene oxide layers, utilizing the confinement effect of bulk traps on Ag^+^ ion migration to effectively suppress random nucleation and overgrowth of conductive filaments, achieving bidirectional gradual conductance tuning. The device stably emulated STDP and “learning-forgetting-relearning” synaptic plasticity even after 200 bending cycles. These studies demonstrate that interface-state trapping directly modifies barrier height, while bulk traps constrain ion migration paths to improve uniformity [35,36], together providing a physical foundation for the design of highly uniform, low-energy flexible memristors.

### 2.3. Ferroelectric Tunnel Junctions and Phase-Change/Mott Memristors

In addition to the aforementioned mechanisms, ferroelectric tunnel junctions (FTJs) and phase-change/Mott-type memristors exhibit significant potential for constructing high-performance flexible neuromorphic devices, owing to their unique physical characteristics.

Ferroelectric tunnel junctions utilize an ultrathin ferroelectric layer as a tunneling barrier; by switching the ferroelectric polarization direction with an external electric field, the height/width of the tunneling barrier is modulated, giving rise to a giant electroresistance (TER) effect. Their typical structure is a metal–ferroelectric–metal (MFM) or metal–ferroelectric–semiconductor (MFS) sandwich configuration, offering fast response, high endurance, and low power consumption, and the resistance state is directly linked to the polarization state, facilitating multilevel storage and synaptic weight updates [37,38]. Two main strategies have been developed to impart flexibility to FTJs. One is the thin-film transfer technique based on water-soluble sacrificial layers; for example, Luo et al. [38] etched away a Sr_3_Al_2_O_6_ (SAO) sacrificial layer to lift off high-quality single-crystalline BaTiO_3_ (BTO)/La_0.7_Sr_0.3_MnO_3_ (LSMO) heterostructures from rigid substrates and transferred them onto flexible PET substrates, achieving over 500% TER effect in the flexible FTJ even under bending. The other is in situ growth directly on high-temperature-resistant flexible substrates such as mica; for example, Sun et al. [37] constructed an MFS-type FTJ on a mica substrate with Au/Ti/ZnO/BiFeO_3_ (BFO)/SRO/BTO/Mica, utilizing an ultrathin BFO (~1.2 nm) barrier layer to achieve multilevel resistance states and low nonlinearity (−0.24) for synaptic plasticity. This device achieved 92.8% accuracy in MNIST handwritten digit recognition simulations, and the switching performance remained stable after 10^3^ bending cycles. Furthermore, Hf_0.5_Zr_0.5_O_2_ (HZO)-based FTJs have also attracted considerable attention. Boynazarov et al. [39] employed a similar transfer technique to transfer HZO/LSMO/SAO heterostructures onto PET substrates, utilizing the oxygen vacancy migration mechanism at the HZO/LSMO interface to achieve bipolar resistive switching, with good bending stability and 94.2% accuracy in handwritten digit recognition.

Phase-change and Mott memristors exploit abrupt changes in the lattice or electronic state of materials. Phase-change memristors are typically based on chalcogenide compounds (e.g., Ge_2_Sb_2_Te_5_), which undergo reversible transitions between crystalline (low-resistance) and amorphous (high-resistance) states under Joule heating or pulsed operation [40,41], offering large resistance ratios but relatively high power consumption. In contrast, Mott memristors utilize the insulator–metal transition (IMT) in strongly correlated electron systems (e.g., VO_2_) to achieve abrupt resistance changes driven by electric field or temperature, featuring ultrafast switching speeds and unique relaxation oscillation characteristics [42]. For flexible applications, Han et al. [43] fabricated flexible Mott memristors with a Pt/VO_2_/Pt structure on polyimide (PI) substrates, exhibiting excellent endurance (>10^7^ cycles) and bending stability (radius ~5 mm). Notably, a single VO_2_ memristor can be used to construct an artificial spiking photosensor (ASP) that encodes light intensity into spike frequency across a broad spectral range (405–808 nm), providing a minimalist hardware foundation for spiking neural networks (SNNs). The same team also built a flexible artificial spiking warm receptor that mimics biological thermoreceptors by exploiting the temperature sensitivity of VO_2_ memristors [42]. These studies indicate that VO_2_-based Mott memristors can not only implement synaptic functions but also emulate neuronal information encoding behavior, making them strong candidates for constructing highly integrated, low-power flexible sensing–memory–computing systems.

### 2.4. Multi-Mechanism Synergy and Kinetic Effects

Flexible memristors often involve the synergistic interplay of multiple resistive switching mechanisms, and the analysis of their switching kinetics is crucial for understanding device behavior and optimizing synaptic performance [44]. The synergy between different mechanisms is primarily manifested in three aspects: functional layer design, interface engineering, and material composite strategies. By constructing heterostructures or employing doping modification, processes such as ionic migration, charge trapping, and thermal effects can be coupled. This suppresses the randomness of CF growth, extends state retention time, or enables multimodal switching behavior. The core of this synergy lies in exploiting the complementarity of various physical processes to achieve high linearity, low power consumption, and excellent uniformity, which are difficult to realize simultaneously with a single material or structure. In flexible HfO_x_/TaO_x_ bilayer memristors, Saleem et al. [30] utilized the oxygen-vacancy-rich nature of the TaO_x_ layer to guide the preferential nucleation and rupture of CFs within the HfO_x_ layer, achieving gradual switching and good cycle-to-cycle uniformity, thus providing a feasible pathway for emulating highly linear synaptic plasticity. On the other hand, the morphology of CFs in organic memristors directly influences abrupt or gradual switching characteristics. Jung et al. [45] used UV-ozone treatment to tune the conductivity of the poly(3,4-ethylenedioxythiophene):poly(styrenesulfonate) (PEDOT:PSS) polymer layer, transforming the filaments from a conventional bridge-type to a cluster-type structure. This not only significantly reduced the device leakage current but also achieved highly linear multilevel conductance modulation, offering distinct advantages for flexible neuromorphic systems. Parylene-based memristors exhibit interesting second-order kinetic effects: pre-heating pulses can increase the temperature of the switching layer via localized Joule heating, significantly shortening the response time of subsequent switching pulses. This dynamic behavior, governed by thermal history, provides a physical basis for emulating spike-rate-dependent plasticity (SRDP) in biological synapses [40]. In Nb_2_O_5_-based flexible memristors, Prajapati et al. [28] discovered that gradual switching dominated by oxygen-vacancy CFs at low voltages and threshold switching induced by the insulator-to-metal transition at high voltages can coexist in the same device. This dual-mode synergistic mechanism can be used both for constructing artificial synapses and as a selector to suppress sneak-path currents in crossbar arrays. Furthermore, environmental factors can also synergize with switching mechanisms. For instance, water and Na^+^ ions can modulate the formation of oxygen-vacancy conductive pathways in TiO_x_-based memristors, altering the operating voltage and ON/OFF ratio, offering new perspectives for designing memristive devices suitable for wearable or implantable environments [46]. Carbon-coated MoSSe heterostructure memristors combine the synergistic effects of vacancy defects and a carbon skeleton, achieving not only a high ON/OFF ratio exceeding 10^5^ and excellent bending stability but also successfully emulating STDP learning rules with a microsecond-scale time window, demonstrating the potential of multi-mechanism synergy in high-speed neuromorphic computing [47].

In summary, the synergistic design of multiple switching mechanisms can not only overcome the inherent limitations of a single mechanism regarding switching linearity, energy consumption, and reliability, but also endow devices with multifunctional capabilities such as self-rectification, environmental adaptability, and temporal information encoding. By integrating the advantages of different physical processes within the same device, researchers can achieve higher computational accuracy and enhanced environmental robustness while maintaining low power consumption. Therefore, a deep understanding and proactive design of the synergy and kinetic processes of multiple resistive switching mechanisms are critically important for guiding the development of high-performance, multifunctional, and environmentally robust flexible memristors.

## 3. Materials for Flexible Memristors

**Figure 2:**
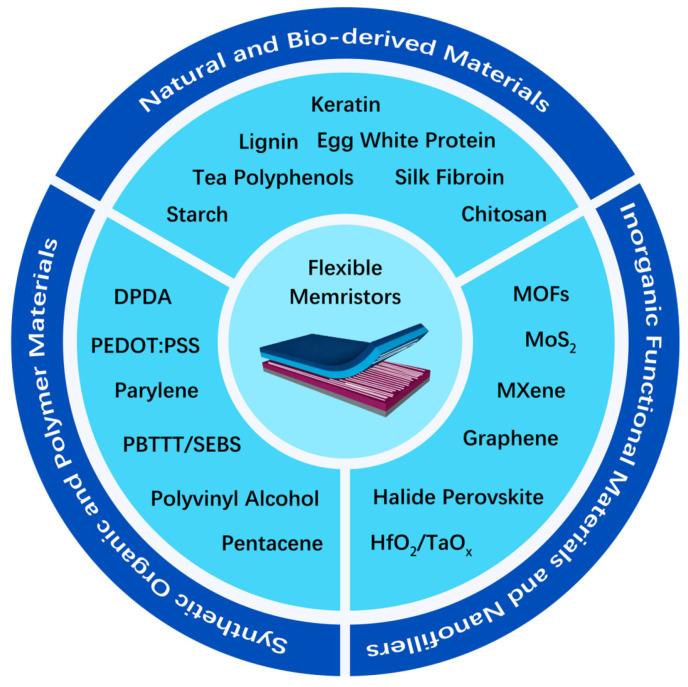
Raw materials used for flexible memristors.

Flexible neuromorphic devices impose stringent requirements on functional layer materials, including low power consumption, high mechanical flexibility, and stable resistive switching characteristics. Although conventional rigid inorganic materials exhibit excellent performance, they fall short of meeting the demands of flexible and wearable applications. To address this, researchers have developed natural and bio-derived materials, synthetic organic and polymer materials, and inorganic functional materials. Among these, two-dimensional materials (e.g., graphene, transition metal dichalcogenides) and MXenes exhibit unique advantages owing to their atomic-scale thickness, tunable surface chemistry, and intrinsic flexibility [1,48]. This section reviews the research status, key properties, and representative applications of the aforementioned material systems, and discusses current challenges and future directions (Figure 2).

### 3.1. Natural and Bio-Derived Materials

Natural and bio-derived materials (e.g., proteins, polysaccharides) possess good biocompatibility, biodegradability, and intrinsic flexibility, making them ideal candidate systems for constructing green, wearable, and implantable neuromorphic devices [16]. But in fact, most natural materials suffer from problems such as weak mechanical properties, strong hydrophilicity, or poor spinnability, making it difficult to meet the requirements of memristors for stable resistive switching and durability. Therefore, strategies to modulate their microstructure, such as molecular crosslinking, nanocomposite filler integration, or blending modification, have become key to enhancing the applicability of this class of materials [49]. Representative material performance is summarized in Table 1.

#### 3.1.1. Protein-Based Materials

Proteins offer advantages for constructing green, flexible, and implantable memristors due to their inherent biocompatibility, degradability, and abundant functional groups. The processability of most proteins is poor, making it difficult to directly form high-quality functional thin films. This stems from their high solution viscosity or insufficient electrical conductivity, leading to poor film uniformity. On the other hand, natural proteins generally exhibit weak mechanical strength and strong hydrophilicity, making them prone to swelling or even dissolving in humid environments. So modification strategies such as incorporating functional fillers or chemical crosslinking are necessary to enhance their film-forming ability, mechanical properties, and water resistance [57]. Among the various protein-based materials, silk fibroin, keratin, and egg white albumin have garnered widespread attention due to their unique molecular structures and electrical activities.

Silk fibroin (SF), a protein extracted from natural silk, exhibits excellent antioxidant and water vapor barrier properties. However, strong hydrogen bonding between SF molecular chains results in poor solution spinnability and low film quality. Zhang et al. [50] improved film quality by blending SF molecular chains with easily spinnable polymers such as poly(ethylene oxide) (PEO). This SF-based memristor stably emulated synaptic plasticity behaviors such as excitatory postsynaptic current (EPSC) and paired-pulse facilitation (PPF) even under bending conditions (with a bending radius as low as 5 mm). This improvement is attributed to the PEO blending modification, which preserves the intrinsic flexibility of the protein while meeting the reliability requirements of neuromorphic computing devices.

**Figure 3:**
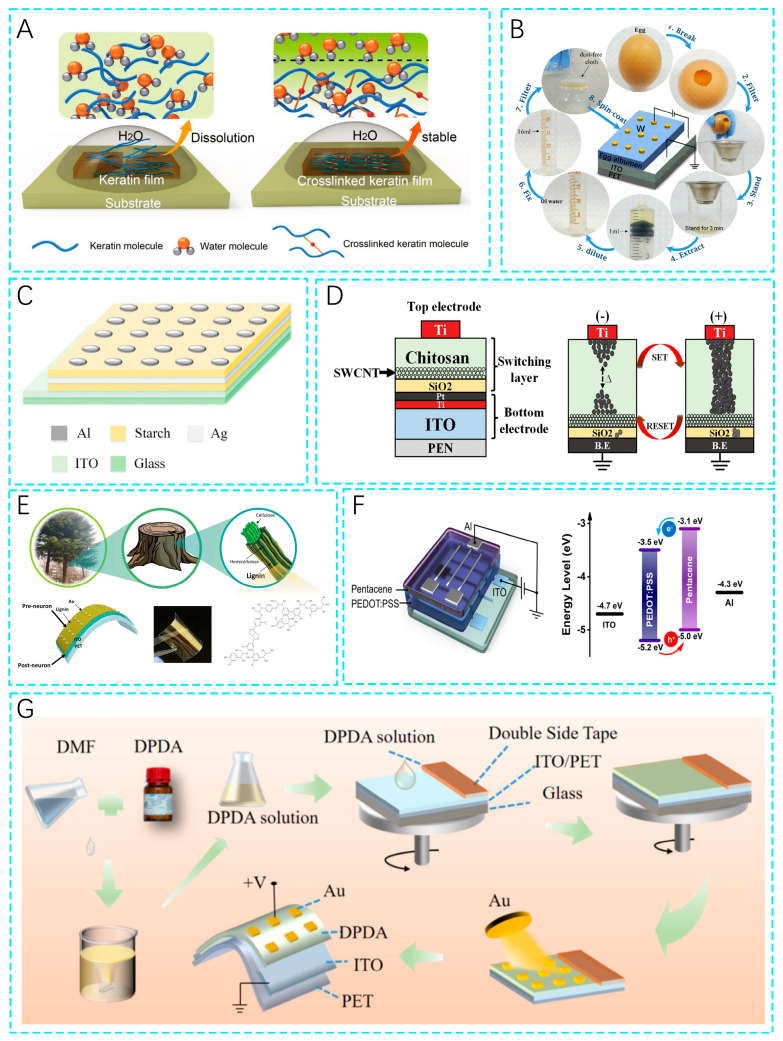
Natural and synthetic materials: (**A**) Schematic structures of water-soluble keratin film and water-insoluble crosslinked keratin film [51]. (**B**) Fabrication process flow and device structure schematic of the W/egg white/ITO/PET memristor [52]. (**C**) Structural schematic of the Al/starch/Ag/starch/ITO memristor [54]. (**D**) Cross-sectional view of the chitosan-single-walled carbon nanotube composite memristor and simplified schematic of the mechanism of conductive filament generation and rupture [55]. (**E**) Device structure schematic of the Al/lignin/ITO/PET flexible artificial synapse and the chemical structure of lignin [56]. (**F**) Device structure schematic of the ITO/PEDOT:PSS/pentacene/Al memristor and energy level alignment diagram of the functional materials [58]. (**G**) Fabrication process flow of the Au/DPDA/ITO device [59].

Keratin, a structural protein derived from natural sources such as wool and feathers, is rich in cysteine and active side groups, endowing it with the ability to coordinate with metal ions. However, natural keratin exhibits strong water solubility and weak mechanical properties. Through photochemical crosslinking modification, the intermolecular forces within keratin can be significantly enhanced. The modified keratin memristor operated stably even after 48 h of immersion in water and maintained switching characteristics at a bending radius as small as 1.2 mm. Shi et al. [51] utilized chemically crosslinked wool keratin to construct flexible artificial synapses, successfully emulating synaptic functions such as long-term potentiation/depression (LTP/LTD) and spike-timing-dependent plasticity (STDP), and achieved a pattern learning accuracy of 95.8% under noisy conditions (Figure 3A).

Egg white albumin exhibits high transparency, good biocompatibility, and intrinsic flexibility, making it suitable for green flexible neuromorphic devices. Yan et al. [52] fabricated a flexible and transparent memristor with a W/egg white/indium tin oxide (ITO) structure using an inert tungsten electrode. This device achieved a transition from short-term to long-term plasticity and emulated the conversion from paired-pulse facilitation to paired-pulse depression by adjusting the pulse amplitude (Figure 3B). Furthermore, doping egg white with functional nanomaterials can effectively enhance the device switching ratio. Wang et al. [60] incorporated gold nanoparticles (AuNPs) into egg white albumin. Leveraging the Coulomb blockade effect of the AuNPs to increase the switching ratio, the fabricated Al/egg white:AuNPs/ITO flexible memristor achieved multi-level nonvolatile storage and rich advanced synaptic functions.

#### 3.1.2. Polysaccharides and Other Biomass

Polysaccharides and other biomass materials (e.g., starch, chitosan, tea polyphenols, lignin) possess abundant functional groups such as hydroxyl and amino groups along their molecular chains, providing a natural structural basis for crosslinking, blending, and ion modulation. This makes them ideal active layer materials for constructing green flexible memristors. Starch is a widely available and low-cost natural polysaccharide. Constructing a three-dimensional network structure through physical or chemical crosslinking can effectively improve the comprehensive performance of starch-based materials. Xie et al. [54] fabricated a threshold memristor with an Al/starch/Ag/starch/ITO structure using a starch-glycerol-hydrogel as the dielectric layer. The device achieved an ON/OFF ratio as high as 2 × 10^7^ and a stable threshold voltage, and successfully emulated key functions of auditory neurons, including spike firing, spatial and temporal summation, and gain modulation (Figure 3C). Chitosan is a cationic natural polymer. The high density of charges along its molecular chains leads to poor spinnability, so it is often blended with PEO or poly(vinyl alcohol) (PVA) to improve film-forming properties. The chitosan-based memristor fabricated by Min et al. [55], through the modulation of ion migration, successfully emulated synaptic behaviors such as paired-pulse facilitation (PPF) and spike-timing-dependent plasticity (STDP) (Figure 3D). In addition, tea polyphenols, as small-molecule active biomass, contain pyrogallol and catechol groups that can reduce Ag^+^ to promote the growth of conductive filaments, thereby enabling reversible volatile resistive switching [49]. Park et al. [56] used lignin as a raw material to prepare flexible memristors via a simple solution-processing method, and successfully emulated various synaptic functions including short-term plasticity (STP), long-term plasticity (LTP), and spike-rate-dependent plasticity (Figure 3E).

#### 3.1.3. Challenges Related to Batch-to-Batch Variability and Reproducibility

Despite the significant advantages of natural and bio-derived materials in biocompatibility, sustainability, and intrinsic flexibility, their practical application faces a non-negligible bottleneck-batch-to-batch consistency and device reproducibility. Unlike chemically synthesized inorganic or polymer materials, natural materials (such as silk fibroin, keratin, egg white, lignin, chitosan, etc.) are subject to biological individual differences, extraction processes, seasonal environmental factors, etc., leading to significant batch-to-batch variations in molecular weight distribution, functional group density, and microstructure. For example, the content of endogenous metal ions (Na^+^, K^+^, Fe^3+^, etc.) in egg white albumin varies with poultry feeding conditions, directly affecting the randomness of conductive filament nucleation and the dispersion of switching parameters [52,60]; the chemical composition of lignin varies greatly depending on plant species and separation processes, thus constraining the controllability of its Joule-heating-induced carbonization behavior [56]. Moreover, within the same batch, pinhole defects and film thickness non-uniformities during solution casting further exacerbate device-to-device performance dispersion [55]. These issues lead to a lack of cross-batch statistical comparability of key metrics such as switching voltage, ON/OFF ratio, and cycling endurance reported in the literature, posing fundamental difficulties for device modeling, process standardization, and large-scale fabrication. To address this challenge, researchers are attempting to mitigate the inherent fluctuations of natural raw materials through chemical crosslinking modification (e.g., photocrosslinking of keratin [51]), blending strategies (e.g., silk fibroin/PEO blend [50]), and nanocomposite incorporation (e.g., chitosan/SWCNT [55]), thereby improving the structural uniformity of the functional layer to a certain extent. However, to fundamentally solve the batch-to-batch variation problem, it is still necessary to develop standardized raw material extraction and purification procedures, establish stringent raw material characterization indicators, and explore alternative approaches based on recombinant proteins or bio-inspired synthetic peptides, so as to effectively bridge the gap from laboratory exploration to engineering applications for natural material systems.

### 3.2. Synthetic Organic and Polymer Materials

Although synthetic organic and polymer materials are inferior to inorganic materials in terms of electrical stability, their unique solution processability, intrinsic flexibility, and low-cost advantages make them ideal choices for large-area flexible neuromorphic devices. Through strategies such as crosslinking, doping, or interface engineering, the growth dynamics of conductive filaments and ion migration behaviors can be effectively modulated, thereby enabling diverse switching functions including volatile and nonvolatile behaviors [61,62]. Representative material performance is summarized in Table 2.

#### 3.2.1. Non-Degradable Polymers

Non-degradable polymers still hold an important position in the field of flexible memristors due to their excellent chemical inertness, thermal stability, and process compatibility, among which Parylene (PPX) is one of the most representative materials. Although PPX is conventionally used as an insulating passivation layer or protective coating in traditional microelectronics due to its high dielectric strength and low leakage current, in memristor applications it serves as the active switching layer. Its amorphous structure provides free volume and thermal stability that support the migration of active metal ions and the controlled formation/rupture of conductive filaments, thereby enabling electrochemical metallization-type resistive switching [40]. Based on this mechanism, Yuklyaevskikh et al. [19] fabricated a Cu/PPX/ITO device by vapor-depositing a PPX layer on a flexible PEN substrate and sputtering a Cu top electrode. They systematically evaluated the influence of mechanical bending on resistive switching characteristics: under constant bending mode, the device maintained stable bipolar switching at a bending radius as low as 0.5 cm, with no significant degradation in Set/Reset voltages or HRS/LRS resistances, and achieved at least 12 distinguishable multilevel resistance states, with intermediate states retaining their values for over 300 s under bending. In the stress-free mode, the electrical characteristics remained nearly unchanged, and the device successfully emulated spike-timing-dependent plasticity (STDP), with conductance changes showing potentiation (up to 30%) for positive delays and depression (up to 50%) for negative delays, consistent with the Hebbian learning rule of biological synapses [19]. To address the high power consumption commonly faced by organic memristors, Chen et al. [63] inserted a CVD-grown graphene layer as a blocking barrier between the Al electrode and PPX. They found that the intrinsic nanopores of CVD graphene (average pore size ~10 nm, density ~1 per μm^2^) effectively confined the metal ion injection paths, resulting in thinner and fewer conductive filaments. Compared to devices without the graphene layer, this structure reduced the reset current from an average of 2.97 mA to 63 μA (approximately 47-fold reduction), and the programming power consumption from about 2.1 mW to below 150 μW, while maintaining >10^4^ s retention and good switching uniformity; conductive atomic force microscopy characterization further confirmed this filament confinement mechanism [63]. Matsukatova et al. [40] systematically studied the switching kinetics and second-order effects of Cu/PPX/ITO and Ag/PPX-Ag/ITO structures, finding that pre-heating pulses could raise the temperature of the PPX matrix through local Joule heating, significantly shortening the subsequent switching time (e.g., from ~20 ms to ~3 ms for a 4.4 V pulse) and increasing the resistance change amplitude by an order of magnitude. Based on this thermally assisted second-order effect, they successfully implemented STDP using non-overlapping symmetric spikes composed of a short high-amplitude pulse (1 ms, 1.1 V) and a long low-amplitude pulse (20 ms, 0.4 V), with a time window width of about 200 ms, matching the millisecond time scale of biological synapses, and the modulation amplitude increased with pre-pulse amplitude, providing a more biologically realistic synaptic plasticity scheme for low-power spiking neural networks [40]. These studies demonstrate that PPX, owing to its moderate permeability to metal ions, stable amorphous structure, and good thermal response, can be transformed from a conventional dielectric into a fully functional memristor active layer, exhibiting unique advantages in flexible, low-power, and plastic neuromorphic computing.

In addition to PPX, non-degradable polymers such as PMMA and PEDOT:PSS are also widely studied in flexible neuromorphic memristors due to their excellent solution processability and process compatibility. Although these materials are often mistakenly considered degradable, they are not environmentally degradable polymers; however, their intrinsic flexibility and low-temperature film formation make them important candidate systems for flexible devices. Composite functional layers based on PMMA can be prepared by low-temperature solution methods, demonstrating excellent synaptic plasticity emulation capabilities. Wang et al. [64] dispersed ZnO nanosheets in a PMMA matrix and spin-coated the composite at an ultra-low temperature of 80 °C to fabricate Ag/ZnO NS@PMMA/Pt flexible memristors. Utilizing the charge trapping/detrapping mechanism induced by oxygen vacancies enriched on the ZnO nanosheet surfaces, rather than traditional conductive filament paths. They achieved highly linear and symmetric conductance updates with operating voltages as low as ±0.4 V, and stable performance after 1000 cycles at a bending radius of 5 mm. Based on this device, a neural network achieved 97.7% accuracy on MNIST handwritten digit recognition, approaching the theoretical limit, strongly validating the potential of PMMA-based composite systems for high-precision neuromorphic computing. Meanwhile, PEDOT:PSS has attracted much attention due to its tunable conductivity and excellent flexibility. However, it should be particularly noted that PEDOT:PSS is essentially a mixed ionic-electronic conductor: the PEDOT chains provide hole-transport-dominated electronic conduction pathways, while the sulfonate groups on the PSS segments impart cation exchange capability (e.g., H^+^ or metal ion migration). This means that the resistive switching behavior and OFF-state leakage current are actually contributed by both electronic and ionic carriers, and their relative weights are highly dependent on the material formulation and applied electric field conditions. The relatively high intrinsic conductivity of PEDOT:PSS (especially after optimization with additives such as ethylene glycol) is beneficial for reducing the operating voltage in the ON state, but inevitably introduces significant leakage current in the OFF state, which becomes a key factor limiting the ON/OFF ratio and static power consumption. Luo et al. [58] used a PEDOT:PSS/pentacene type-II heterojunction device, leveraging band alignment to reduce the operating voltage to ±1.0 V and the operating current to below 10 µA. The PEDOT:PSS layer acts as a hole injection layer and conductive matrix, and the resistive switching behavior originates from charge trapping/detrapping caused by the redox conversion, which is a typical electron-conduction-dominated mechanism. However, the MNIST recognition accuracy of this device dropped from 92.6% to 89.0% after 1000 bending cycles, which Luo et al. attributed to the further deterioration of leakage paths due to microcracks or interface degradation under mechanical deformation, exacerbated by the high intrinsic conductivity of the PEDOT:PSS layer. In contrast, the pure pentacene thin-film memristor studied by Han et al. [65] completely avoids the mixed conduction issue, relying on hole trapping/detrapping in the p-type organic semiconductor to achieve coexistence of digital and analog switching, successfully emulating “learning-forgetting-relearning” behavior on a flexible PET substrate with 93.8% MNIST accuracy. In summary, leakage current management in PEDOT:PSS-based devices requires a clear distinction between the contributions of electronic and ionic conduction, and targeted suppression of OFF-state leakage through band engineering or introduction of trap sites, while also paying attention to the influence of environmental humidity on PSS^-^ ionic conduction. This provides important optimization directions for the application of PEDOT:PSS in low-power flexible neuromorphic computing.

Organic small-molecule systems also demonstrate ultra-low energy consumption and excellent flexibility. DPDA (1,4-diphenylbutadiyne)-based memristors utilize the torsional effect of the molecular conjugated bridge under an electric field to achieve resistive switching, rather than relying on traditional conductive filament formation and rupture, thus inherently offering lower operating currents and higher controllability. Under single-pulse excitation, the energy consumption of this device is as low as 20.9–29.9 fJ, and it stably emulates LTP/LTD even after 1000 bending cycles, showing excellent resistance to mechanical fatigue [59]. To further suppress the random growth of conductive filaments and improve device uniformity, Dong et al. [69] have proposed a “high-efficiency distribution conversion bridge” strategy, dispersing the organic semiconductor poly [2,5-bis(3-tetradecylthiophen-2-yl)thieno [3,2-b]thiophene] (PBTTT) in a styrene-ethylene/butylene-styrene block copolymer (SEBS). Utilizing the electrostatic interaction between sulfur atoms in PBTTT and Al^3+^ ions, ion migration is guided along predefined paths. When the PBTTT content is 10 wt%, the device exhibits nonvolatile Flash-type memory characteristics with power consumption as low as 10^−7^–10^−10^ W and extremely small switching voltage fluctuations over 500 write/erase cycles. When the PBTTT content is increased to 50 wt%, the device switches to volatile dynamic behavior, achieving dual-mode memristive functionality within the same material system. In addition, by pre-forming initial conductive filaments through electroforming in a crosslinkable polymer (such as PVCi) and then locking the localized ion migration paths via UV crosslinking, the random growth of filaments is effectively suppressed, reducing switching voltage fluctuations by about fourfold, and stable resistive switching is maintained after 300 cycles at a bending radius of 5 mm [70].

Non-degradable polymer-based memristors, with their solution processability and intrinsic flexibility, have made significant progress in neuromorphic computing. Systems such as PPX, PMMA, PEDOT:PSS, DPDA small molecules, and PBTTT/SEBS blends have respectively achieved bending-stable multilevel resistance states, highly linear conductance updates, digital/analog dual-mode switching, femtojoule-level energy consumption, and controllable ion migration. Strategies such as molecular conformation design, blending, and crosslinking confinement have effectively improved device uniformity and bending robustness. However, interface reliability, environmental compatibility, and large-area consistency remain major bottlenecks for practical applications, requiring multi-strategy synergistic optimization.

#### 3.2.2. Degradable Polymers

Water-soluble synthetic polymers such as poly(vinyl alcohol) (PVA), owing to their excellent water dispersibility, biocompatibility, and controllable degradation, exhibit unique advantages in green transient electronics and implantable/wearable neuromorphic devices. These polymers are rich in polar groups such as hydroxyl groups, which impart intrinsic hydrophilicity and water solubility, and their degradation products are non-toxic small molecules, aligning with the development needs of low-environmental-impact electronics. However, strong hydrophilicity also leads to poor moisture resistance, insufficient mechanical strength, and non-uniform film formation, limiting their long-term stable operation in complex mechanical environments. To address these challenges, researchers have employed strategies such as crosslinking, blending, or incorporation of nanofillers to effectively modulate the charge trap distribution and ion migration behavior while maintaining degradability, achieving synergistic optimization of switching performance and mechanical flexibility. Xiong et al. [66] blended graphene oxide (GO) with PVA, utilizing the strong hydrogen-bonding network formed between the oxygen-containing functional groups of GO and PVA chains to fabricate ITO/PVA-GO/ITO flexible memristors. These devices exhibited forming-free operation, threshold voltages as low as ±0.2 V, SET power consumption of only about 0.5 μW, stable resistance states after repeated bending, and successful emulation of Pavlovian associative learning behavior. These results demonstrate that PVA-based material modification strategies provide a feasible path for constructing low-power, degradable flexible neuromorphic systems, although long-term environmental stability and interfacial compatibility with biological tissues still require further optimization.

Poly(ethylene oxide) (PEO), as another water-soluble semi-crystalline polymer, is also widely used in flexible memristor research due to its excellent film-forming ability, flexibility, and abundant ether oxygen groups (which provide ion transport channels). However, PEO’s high crystallinity, relatively low room-temperature ionic conductivity, and strong hydrophilicity (leading to moisture absorption and deformation in humid environments) limit its standalone application in stable neuromorphic computing [50]. To overcome these limitations, researchers have employed strategies such as nanocomposite incorporation or heterostructure design to endow devices with volatile switching and bio-inspired sensing functions while maintaining PEO processability. For example, Deb et al. [71] embedded CuS nanoparticles in a PEO matrix, utilizing the dynamics of PEO chain segments to regulate the migration rate of Cu^+^ ions, achieving for the first time a copper-based diffusive volatile memristive switch in a polymer system. The device exhibited an ON/OFF ratio as high as 4 × 10^3^, a low threshold voltage of about 0.5 V, and a relaxation time of 400 μs, and successfully emulated the threshold response, relaxation, and sensitization behaviors of an artificial nociceptor. Lu et al. [68] constructed a flexible memristor based on a bilayer structure of semiconducting single-walled carbon nanotubes (s-SWCNTs) and PEO:LiClO_4_, where Li^+^ and protons in PEO mimic biological Na^+^/K^+^ functions, achieving bidirectional synaptic plasticity through voltage modulation: small voltages (<1 V) induce synaptic potentiation, while higher voltages (>1.4 V) trigger synaptic inhibition. This work systematically emulated the entire “threshold-protection-damage-recovery/irreversible damage” process of pain perception for the first time in an artificial synaptic device, with nearly no performance degradation at a bending radius of about 6 mm.

### 3.3. Inorganic Functional Materials and Nanofillers

Inorganic functional materials (metal oxides, two-dimensional materials, perovskites, etc.) have demonstrated reliable resistive switching behavior in rigid memristors owing to their excellent electrical properties, high thermal stability, and mature fabrication processes. Through low-temperature atomic layer deposition, sputtering, or solution methods combined with nanostructure engineering, researchers have achieved the integration of high-quality inorganic functional thin films on flexible substrates, enabling stable operation even under repeated bending or stretching conditions [72]. Representative material performance is summarized in Table 3.

#### 3.3.1. Metals and Metal Oxides

**Figure 4:**
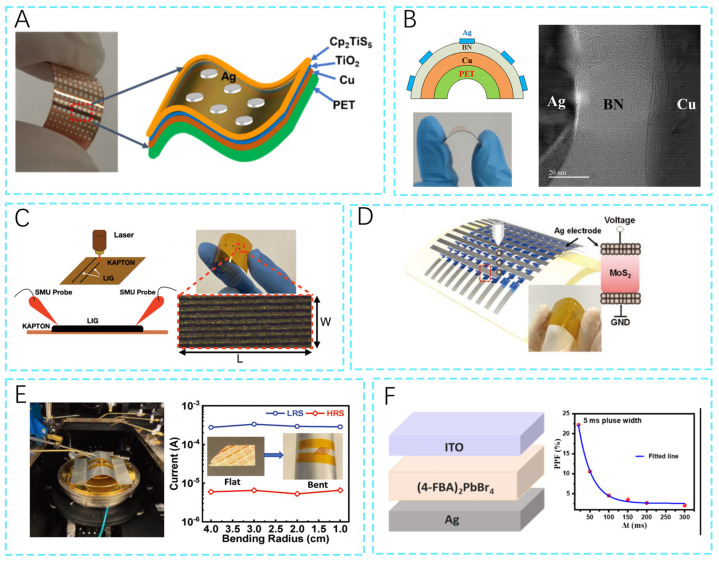
Inorganic functional materials: (**A**) Optical photograph and structural schematic of the flexible PET/Cu/TiO_2_/Cp_2_TiS_3_/Ag device [81]. (**B**) Device structure schematic and cross-sectional TEM image of the Ag/h-BN/copper foil memory cell on a PET substrate [87]. (**C**) Process flow schematic of laser-induced formation of closely packed grooves on a polymer substrate [78]. (**D**) Schematic plot of the fully printed Ag/MoS_2_/Ag memristor in a 4 × 4 crossbar structure on flexible polyimide substrate [88]. (**E**) Schematic of the flexible black phosphorus device [79]. (**F**) Schematic of a memristor emulating biological synaptic behavior [80].

Metal oxide memristors mainly rely on the oxygen-vacancy conductive filament mechanism to achieve resistive switching. Common materials include HfO_2_, TaO_x_, TiO_2_, ZnO, and WO_x_ [81,82] (Figure 4A). For example, Saleem et al. [30] employed a TaO_x_/HfO_2_ bilayer structure, utilizing the difference in oxygen vacancy concentration between the two layers to form confined “hourglass-shaped” conductive filaments, which significantly improved switching uniformity and the linearity of analog resistive switching. Yang et al. [83] grew colorful amorphous Nb_2_O_5_ thin films in situ on flexible Nb foil via anodization. The Ag/Nb_2_O_5_/Nb device achieved an ON/OFF ratio as high as 10^5^ and a data retention capability exceeding six months. Hasina et al. [73] fabricated an Ag/ZnO/ITO memristor on a PET substrate using pulsed laser deposition. By modulating the oxygen vacancy concentration, they successfully emulated both synaptic plasticity and nociceptor functions within a single device.

Other metal oxides such as VO_2_, CeO_2_, and InGaZnO also exhibit excellent synaptic plasticity and bending stability. VO_2_, based on the insulator-metal transition characteristic of Mott memristors, can generate relaxation oscillations under constant current excitation, making it an ideal candidate for implementing spike-encoding artificial receptors in a single device. A Pt/VO_2_/Pt Mott memristor fabricated on a flexible PEN/polyimide (PI) composite substrate exhibited a low threshold voltage (~0.6 V), high cycling endurance (>10^4^ cycles), and excellent bending stability (radius as low as 5 mm). A relaxation oscillator constructed by connecting a single VO_2_ memristor in series with a current source produced output spike frequencies that increased with temperature in the range of 20–40 °C and rapidly dropped to zero above 45 °C, perfectly mimicking the frequency-temperature response characteristics of biological thermoreceptors. Furthermore, using a VO_2_ device with a transparent top electrode, an artificial spiking photosensor integrating light sensing and spike encoding was realized, and a full-spiking electronic retina architecture was constructed based on this device [42,43]. Nanometer-thick CeO_2_-based memristors can achieve dual-mode switching between volatile threshold switching and nonvolatile memristive switching by adjusting the compliance current and film crystallinity, simultaneously emulating the integrate-and-fire function of neurons and the plasticity transition of synapses within the same device [74]. Through interface engineering and composition tuning, these metal-oxide-based flexible memristors can meet the multiple requirements of synaptic biomimetics and mechanical robustness [84,85].

Compared with the aforementioned polycrystalline metal oxides, amorphous oxide semiconductors (AOS) offer unique advantages in flexible neuromorphic memristors due to the absence of grain boundary scattering, the ability to achieve large-area uniform films via low-temperature solution or sputtering processes, and smaller electrical performance degradation under bending deformation. Through compositional gradient design, α-GTO-based memristors can achieve forming-free operation and an ON/OFF ratio as high as 448, with conductance continuously tunable by the Set voltage, providing a physical basis for analog-type synaptic plasticity [75]. Furthermore, hydrogen doping extends the photoresponse of IGZO-based optoelectronic memristors to the green wavelength (505 nm), with pulse energy consumption as low as 0.01 fJ at a read voltage of 0.001 V, and successfully emulates STDP and “faster learning, slower forgetting” cognitive behaviors [76]. Based on a single AOS material system, researchers have constructed a complete neuromorphic computing core including neurons (IGZO TFTs), synapses (GTO memristors), and rectifying diodes (IGZO:H), achieving 94.1% accuracy on MNIST recognition with excellent tolerance to conductance variation (standard deviation 20%) [77]. In addition, indium-free AOS (such as ZnSnO-based systems) maintain mobility of 20–50 cm^2^/Vs while avoiding the scarcity of indium resources; their memristors have achieved >100 stable switching cycles and synaptic functions such as LTP/LTD [86].

#### 3.3.2. Boron Nitride-Based Materials and Carbon-Based Materials

Boron nitride-based memristors offer unique advantages in the field of low-power in-memory computing. Studies have shown that flexible BN nanosheet memristors simultaneously achieve nonvolatile Boolean logic computing and ultra-low-power analog neuromorphic computing (energy consumption per synaptic event of 198 fJ) within a single device [7]. To avoid damage introduced by the transfer process of two-dimensional materials, Wang et al. [87] adopted a transfer-free strategy to fabricate memristors directly on Cu foil on which h-BN thin films had been grown. By changing the top electrode material, they achieved controllable switching between analog-type (LTP/LTD) and digital-type (ON/OFF ratio > 10^6^) resistive switching behaviors (Figure 4B).

Carbon-based materials (e.g., graphene, graphene oxide) exhibit great potential in flexible neuromorphic memristors, owing to their excellent mechanical flexibility, tunable interfacial properties, and good electrical/thermal conductivity. Through structural design, molecular functionalization, or material system optimization, researchers have successfully achieved controllable fabrication of devices ranging from volatile threshold switching to nonvolatile analog/digital dual-mode memristive behavior. Ganeriwala et al. [78] used laser direct writing to produce laser-induced graphene on polyimide in a single step, obtaining volatile memristors that successfully emulated synaptic inhibition and “learning-forgetting-relearning” behavior. Through circuit design, they also realized leaky integrate-and-fire neuron functions (Figure 4C). To address the abrupt switching observed in graphene oxide-based memristors due to excessive conductive filament growth, Li et al. [36] inserted an organic pyridinium salt buffer layer between two graphene oxide layers. This buffer layer effectively modulated Ag^+^ migration, achieving bidirectional gradual conductance modulation and various synaptic functions. Furthermore, Zhang et al. [89] prepared graphene nanosheets with high-density surface phosphate groups via phosphorylation modification. Utilizing the strong coordination between phosphate groups and Ag^+^, they precisely controlled the formation and stability of conductive filaments, obtaining LTP/LTD characteristics with extremely low nonlinearity.

#### 3.3.3. Other Functional Materials

Metals, metal oxides, boron nitride-based materials, and carbon-based materials have already demonstrated excellent resistive switching properties in flexible memristors. In addition, other functional materials (e.g., two-dimensional materials, perovskites, metal–organic frameworks, quantum dots, ferroelectric materials) also exhibit significant advantages in ultra-low power consumption, optoelectronic synergy, and multimodal sensing owing to their unique physical mechanisms. Benefiting from their atomic-scale thickness, high carrier mobility, and intrinsic flexibility, two-dimensional materials (e.g., MoS_2_, MXene, black phosphorus) show unique potential for constructing ultra-low-power flexible neuromorphic memristors. Feng et al. [88] investigated all-printed MoS_2_ memristor crossbar arrays that require no high-temperature annealing, achieving an ON/OFF ratio as high as 10^7^ and switching energy down to the femtojoule level (Figure 4D). Hota et al. [48] and Xu et al. [90] both studied MXene materials, which, owing to their high electrical conductivity and tunable surface functional groups, exhibit excellent endurance and multilevel storage capability at low operating voltages. Kumar et al. [79] focused on black phosphorus, which, due to its high mobility and tunable bandgap, was successfully applied in optoelectronic synapses to achieve light-induced transition from short-term to long-term plasticity (Figure 4E). Han et al. [91] utilized the unique wavelength-dependent bidirectional photoresponse of two-dimensional PtSe_2_ to achieve fully optically driven all-optical synaptic plasticity, opening a new path for flexible neuromorphic vision systems. Halide perovskites (e.g., Cs_3_Bi_2_I_9_) exhibit strong ion migration capability and high defect tolerance, leading to low operating voltage (<0.5 V), high ON/OFF ratio (>10^5^), and good air stability in flexible memristors, successfully emulating synaptic functions such as STDP [25,92,93]. Gayen et al. [80] studied fluorinated two-dimensional Ruddlesden-Popper (RP) perovskite (4-FBA)_2_PbBr_4_. Leveraging the hydrophobicity and dipole effect of C-F bonds, the device reproducibly achieved PPF and LTP/LTD even under bending conditions (Figure 4F). Metal–organic frameworks (MOFs) combine organic flexibility with inorganic porous structures. For example, a Zn-TCPP device exhibited “learning-forgetting-relearning” curves through a charge trapping mechanism, while a ZIF-8/MXene composite achieved an ON/OFF ratio of 10^4^, providing a diverse material platform for flexible neuromorphic computing [3,5,94]. Quantum dots (QDs) and ferroelectric materials exhibit unique advantages in flexible neuromorphic devices due to their confinement effects and polarization switching characteristics. A carbon dot nanoribbon self-assembled memristor maintained a stable switching window after 10^4^ cycles at a bending radius of 10 mm, with a response time of only 5.5 ns [95]. SnS2 quantum dots served as charge trapping centers in a flexible memristor, achieving an ON/OFF ratio as high as 10^6^ and long retention time [96]. For ferroelectric materials, by designing a SrRuO_3_/BaTiO_3_ double buffer layer, (111)-oriented BiFeO_3_ thin films were epitaxially grown on flexible mica, achieving a record-high remanent polarization (~97 μC/cm^2^). Continuous ferroelectric domain switching enabled continuously tunable resistive switching, with an accuracy of ~90% in MNIST recognition simulations [97]. A triboelectric-ferroelectric coupled device based on α-In_2_Se_3_ ferroelectric semiconductor converted tiny mechanical forces (~10 kPa) into high-voltage pulses using contact electrification and electrostatic induction, achieving polarization switching under ultra-low pressure and neuromorphic stress sensing [98].

In summary, the three major material systems—natural and bio-derived materials, synthetic organic and polymer materials, and inorganic functional materials—each possess distinct advantages and have achieved breakthroughs in intrinsic flexibility, solution processability, and electrical performance. However, they all face common challenges such as uniformity, stability, and interface compatibility. In the future, it will be necessary to integrate the advantages of multiple materials and develop green integration processes to promote the practical application of flexible memristors.

## 4. Device Structures and Fabrication Technologies for Flexible Memristors

Constructing high-performance flexible memristors requires a delicate balance between device structure, material selection, and fabrication processes. The core objective is to achieve stable, reliable, and functionally rich resistive switching on flexible substrates, thereby meeting the stringent demands of neuromorphic computing for device uniformity, energy efficiency, and integration density. This chapter starts with fundamental structures, discusses array designs developed for high-density integration, then introduces unconventional structures that break through the limitations of traditional planar configurations, summarizes the low-temperature fabrication technologies that underpin these advances, and finally systematically evaluates the mechanical and environmental stability of flexible devices.

### 4.1. Typical Sandwich Structures and Crossbar Array Designs

**Figure 5:**
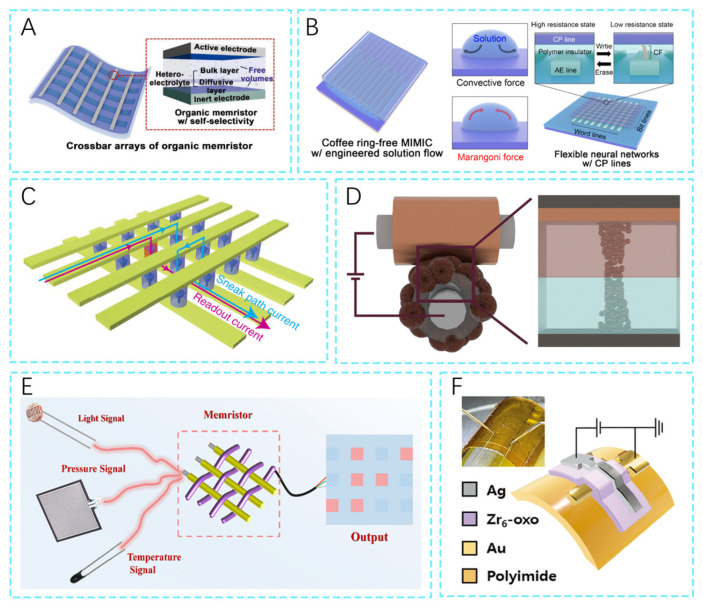
Device structures: (**A**) Schematic diagram illustrating the structure of an organic memristor with self-selectivity for practical applications [101]. (**B**) Neural networks based on the conducting polymer signal lines [100]; (**C**) Schematic of a crossbar array structure [102]. (**D**) Schematic of the RS physical mechanism [103]. (**E**) Structural schematic of a fabric sensing system [104]. (**F**) Schematic of the flexible memristor with the Ag/Zr_6_-oxo/Au device on a polyimide substrate [105].

The most fundamental device structure for flexible memristors is the metal-insulator-metal (MIM) sandwich configuration, where the top electrode, functional layer, and bottom electrode are sequentially stacked on a flexible substrate. The core advantage of this structure lies in its simple fabrication process, which requires no complex high-temperature treatment, and its good compatibility with various flexible substrates (e.g., PET, polyimide). This configuration provides a clear vertical current path for resistive switching and facilitates device performance modulation through electrode and interface engineering, making it the mainstream choice for constructing basic units of flexible neuromorphic computing [99,100,101] (Figure 5A). To achieve high-density information storage and parallel processing, the crossbar array structure is widely adopted in academia. Park et al. [100] employed a coffee-ring-free micromolding in capillaries (MIMIC) technique to fabricate crossbar arrays of conductive polymer signal lines (Figure 5B). In this technique, evaporation-induced outward flow (i.e., convective force) causes solute migration to the edges; meanwhile, the surface tension gradient generated by the addition of co-solvents drives a reverse flow (i.e., Marangoni force). When these two forces are balanced, the solute deposits uniformly, avoiding edge accumulation (the coffee-ring effect), thus obtaining electrode lines with uniform thickness, ensuring the performance uniformity and recognition accuracy of each memristor unit in the array. Li et al. [102] utilized this structure to define each memory cell through orthogonally arranged top and bottom electrodes, effectively suppressing sneak path currents, which makes it a fundamental architecture for large-scale neuromorphic computing hardware (Figure 5C). However, constructing high-performance crossbar arrays on flexible substrates faces special challenges, including low-temperature process limitations, crosstalk issues as array size scales up, and stringent requirements for device uniformity.

Therefore, researchers have proposed solutions from both material engineering and structural design perspectives. On the one hand, low-temperature atomic layer deposition and other techniques enable the direct fabrication of highly uniform functional layer thin films on flexible substrates. Elemental doping (e.g., nitrogen-doped tantalum oxide) achieves “forming-free” switching, simplifying peripheral circuits [18]. Kim et al. [18] systematically studied the effect of nitrogen doping on tantalum oxide-based memristors, finding that with increasing nitrogen doping concentration, oxygen vacancy defects in the film are effectively passivated, and the initial resistance of the device increases significantly, thus enabling stable bipolar resistive switching without the need for a high-voltage electroforming process. More importantly, leveraging the excellent film uniformity of ALD technology, they successfully fabricated a 6 × 6 crossbar array on a flexible PET substrate, with all 36 cells showing good consistency (narrow distribution of ON/OFF ratios), which at the device level reduces the risk of misreading due to cell differences in the array and indirectly alleviates the impact of sneak-path currents on read operation reliability. On the other hand, adopting device structures with intrinsic rectifying characteristics can fundamentally eliminate sneak currents. The all-silicon-based p-Si/SiO_2_/n-Si memristor proposed by Li et al. [102] serves as a paradigm. The key innovation of this device is that the top electrode (p-type heavily doped silicon), the bottom electrode (n-type heavily doped silicon), and the intermediate silicon oxide switching layer together form a built-in diode. Under reverse bias, this pn junction exhibits extremely high resistance (rectification ratio up to 10^5^), so that any sneak-path current through unselected cells (whether lateral paths within the same layer or vertical paths between layers) must pass through at least one reverse-biased cell and is thus effectively blocked. Experimental results show that even under the “worst-case” scenario where all non-target cells are deliberately programmed to the low-resistance state, the array can still correctly read the stored information. Furthermore, by alternately stacking p-type and n-type silicon nanowires, this work achieved multilayer 3D crossbar arrays without the need for interlayer isolation layers, further verifying the effectiveness of the built-in rectifying characteristic in suppressing interlayer crosstalk, providing a new path for constructing high-density, low-power 3D neuromorphic computing hardware.

### 4.2. Unconventional Structure Memristors

To meet the demands of emerging applications such as wearable electronics and smart textiles for seamless integration and multifunctionality, researchers have begun exploring unconventional memristors that go beyond traditional planar structures. Jiang et al. [103] synthesized a BiOI/TiO_2_ heterointerface on carbon fibers via a hydrothermal method. Using the fiber crossover points as natural switching units, they constructed fiber-based memristors that can be directly woven into fabrics, forming distributed computing nodes and integrating with sensors to build intelligent gesture recognition systems (Figure 5D). This fabric sensing system design not only endows electronic textiles with excellent flexibility and breathability but also enables in situ signal processing and computation within smart clothing [104]. Furthermore, Ding et al. [106] demonstrated that by modulating the MoS_2_ functional layer with copper ions, switching behavior can be tuned from volatile to nonvolatile, simultaneously achieving neuromorphic computing and intelligent electromagnetic protection. In addition, Guan et al. [104] used electrophoretic deposition to fabricate a porphyrin-based functional layer on silver fibers, obtaining textile memristors with ultra-low power consumption (456.52 fJ) and an ultra-high ON/OFF ratio (~5 × 10^7^), and emulated multi-sensory cooperative recognition (Figure 5E). The development of these unconventional structures marks the evolution of memristors from single electronic components toward multifunctional, system-integrated intelligent platforms.

### 4.3. Fabrication Technologies

Flexible substrates (e.g., PET, PEN, polyimide) generally cannot withstand high temperatures (typically requiring processing below 200 °C), which conflicts with the high-temperature crystallization processes traditionally required for metal oxide thin films [107]. Therefore, developing low-temperature or even room-temperature fabrication technologies is a key prerequisite for the practical application of flexible memristors.

Physical vapor deposition (PVD) and atomic layer deposition (ALD) are common vacuum techniques for preparing high-quality oxide thin films at low temperatures. Kim et al. [18] employed an ALD process at 150 °C to directly deposit N:TaO_x_ thin films on PET substrates. By passivating oxygen vacancies, they achieved forming-free and analog-type resistive switching. Similarly, Saleem et al. [30] used PVD to fabricate HfO_x_/TaO_x_ bilayer structures, achieving stable and linear synaptic plasticity by modulating the oxygen vacancy gradient. These low-temperature vacuum techniques ensure high-quality growth of functional layers on flexible substrates and serve as the cornerstone for realizing high-performance devices.

In addition to developing low-temperature deposition processes themselves, the compatible integration of flexible memristors with complementary metal-oxide-semiconductor (CMOS) technology is a key step toward practical neuromorphic systems, because such integration can organically combine high-performance computing backend circuits with emerging memory devices. However, this integration faces an inherent process thermal budget conflict: the thermal tolerance limitation of flexible substrates (typically requiring temperatures below 200 °C) not only affects the crystalline quality of functional layer films but also directly constrains compatibility with standard CMOS back-end-of-line (BEOL) processes. Traditional BEOL processes often require temperatures exceeding 400 °C for steps such as interlayer dielectric deposition and contact annealing [31]. To overcome this bottleneck, researchers have developed two main strategies. One is to use low-temperature BEOL-compatible processes to monolithically integrate memristor functional layers in 3D on top of prefabricated CMOS wafers. For example, MOCVD-grown multilayer MoS_2_ was wet-transferred onto the metal interconnect layer of a 350 nm CMOS chip, and then nanoscale cross-point devices were defined by electron-beam lithography, successfully achieving forming-free, nonvolatile bipolar switching with a Set voltage as low as ~0.23 V and cycle-to-cycle variation (CV) below 6.7% in a 1T1R configuration; the integrated transistor simultaneously served as a selector and current limiter, significantly improving array uniformity [31]. This work verified that 2D material memristors can be directly built on top of active circuits without damaging underlying CMOS performance. The other strategy is to use fully CMOS-implemented memristor emulators. Although they are not nanoscale physical switches, they provide a complementary path for prototyping neuromorphic algorithms in standard foundry processes. A representative example is a reconfigurable array containing 4096 nonvolatile memristor emulators implemented in a 0.18 μm CMOS process, where each cell uses a voltage differential current conveyor transconductance amplifier (VDCCTA) to emulate the flux-dependent conductance characteristics of a physical memristor, achieving a conductance tuning range of more than two orders of magnitude (G_max_/G_min_ = 192) and MHz-level operating frequencies, with core power consumption of only 1.16 mW [23]. This platform is not subject to the variability and endurance limitations of experimental resistive switching devices and has been successfully used for hardware verification of adaptive learning tasks such as echo state network power load forecasting [23,108]. These advances show that achieving true CMOS compatibility requires co-design of material selection, device structure, and low-temperature processes, thereby leveraging the functional advantages of emerging memristive technologies while making full use of the mature CMOS infrastructure, paving the way for building flexible, energy-efficient neuromorphic computing systems [109].

Meanwhile, solution-based methods and all-printing technologies (e.g., spin coating, inkjet printing, aerosol jet printing) allow for device fabrication at even lower temperatures (even room temperature) and are more suitable for large-area, low-cost production [72]. For example, all-printed MoS_2_ memristors on polyimide substrates achieved femtojoule-level switching energy and successfully emulated various synaptic functions [88]. Solution-processed RP perovskites and Bi_2_S_3_ nanostructures also exhibit good flexibility and synaptic characteristics [72,110]. These low-temperature solution strategies open up feasible pathways for constructing low-cost, wearable neuromorphic computing systems on plastic substrates.

### 4.4. Flexible Substrates and Mechanical Stability Testing

The practical application of flexible memristors, especially in wearable and implantable devices, requires them to maintain stable electrical performance under repeated bending, folding, and even stretching mechanical environments. Evaluation of device mechanical stability is important, including the selection of flexible substrates, mechanical deformation testing, and considerations of environmental and transient characteristics.

#### 4.4.1. Types of Flexible Substrates

The choice of flexible substrate directly affects the mechanical robustness, process compatibility, and application scenarios of memristors. Currently used materials can be classified into four major categories: plastic films, metal foils, single-crystal flakes, and degradable substrates, each with significant trade-offs among thermal budget, surface quality, and cost. Plastic films (PET, PEN, PI) are the most widely used due to their light weight, flexibility, and excellent bending fatigue properties [33,105] (Figure 5F), among which PI can withstand temperatures above 400 °C, compatible with low-temperature crystallization of some oxides, but is costly; PET and PEN are inexpensive but have glass transition temperatures of only about 150 °C and relatively large surface roughness, mainly suitable for solution-based or room-temperature deposition processes. Yuklyaevskikh et al. [19] fabricated Parylene memristors on PEN substrates that stably emulated STDP synaptic plasticity even at bending radii as low as 5 mm, verifying the feasibility of plastic substrates in wearable neuromorphic systems. Metal foils (e.g., Al foil) serve dual roles as substrate and bottom electrode, withstanding temperatures up to 660 °C; Zuo et al. [29] constructed Ag NPs:a-SiC_0.11_H memristors on Al foil that maintained an ultra-high ON/OFF ratio of about 10^7^ after 1000 bending cycles, but the large surface roughness and poor flatness restrict high-density crossbar integration. Mica flakes can support epitaxial growth of ferroelectric oxides [19], but their brittleness and high cost hinder large-scale application. Paper and degradable polymers (PVA, PEO) are uniquely valuable in green transient electronics [20]; Wang et al. [111] used water-soluble conjugated polyelectrolytes to achieve complete dissolution within 20 s in water, suitable for implantable or information-security disposal scenarios, but their poor water/oxygen barrier properties make long-term reliability a bottleneck. Overall, substrate selection is essentially a multi-objective trade-off among temperature resistance, surface quality, mechanical flexibility, and cost. High-performance oxide systems are preferably suited to PI or metal foils, low-cost wearable scenarios to PET/PEN, and transient biomedical applications to degradable materials. In the future, if large-area flexible substrates can be synergistically advanced with low-temperature 3D integration processes, these trade-off dilemmas may be broken, promoting the transition of flexible memristors from single devices to reliable system-level applications.

#### 4.4.2. Mechanical Deformation Testing Methods

Evaluating the mechanical stability of flexible memristors requires systematic mechanical testing to quantify the degree of electrical performance degradation under repeated bending, folding, and even stretching conditions. Currently, there is no unified standardized testing protocol specifically for flexible neuromorphic memristors; existing evaluation methods are mainly borrowed from general specifications for flexible displays and flexible electronics (such as the bending/torsion test guidelines in IEC 6271) [51]. Most research groups use self-built test setups, typically fixing devices on bending fixtures with specific curvatures and performing bending cycle tests (to evaluate performance degradation after repeated bending) and bending radius tests (to quantify in situ electrical response under different curvatures) [112,113,114]. In bending cycle tests, Kim et al. [18] performed 10^4^ bending cycles (radius 2.5 mm) on N:TaO_x_ flexible crossbar arrays, and the devices maintained stable ON/OFF ratios and 24 h data retention after testing. Gosai et al. [112] repeatedly bent ZnO nanosheet devices to a 5 mm radius for 1000 cycles, with no significant degradation in HRS/LRS and only about 12% fluctuation in the nonlinearity factor. Bending radius tests use semi-cylindrical molds with decreasing curvature; Peng et al. [113] found that CdPS_3_ devices maintained stable resistive switching at a 2 mm radius, and Shi et al. [51] further pushed the bending limit of crosslinked keratin-based devices to 1.2 mm. However, significant differences in parameters such as bending radius (5 mm to 1.2 mm), cycle numbers (10^2^ to 10^4^), and measurement methods (in situ or ex situ) across studies make cross-comparison difficult. In the future, it is urgent to establish dedicated mechanical testing specifications for neuromorphic devices, unifying the real-time electrical readout protocols under dynamic bending and the criteria for performance degradation, to improve the reproducibility and engineering reference value of results.

#### 4.4.3. Environmental Stability and Transient Electronics

In addition to mechanical deformation, flexible memristors in practical applications must also cope with complex environments such as humidity and temperature changes. Devices based on lead-free halide perovskites (e.g., Cs_3_Bi_2_I_9_) exhibit stable operation for over 30 days in an unpackaged high-humidity environment (>80% RH) due to their intrinsic hydrophobicity and strong chemical bonds [92]. Transient electronic devices utilize water-soluble materials (e.g., Sr_3_Al_2_O_6_, collagen) to achieve controlled degradation, offering unique value in implantable medical devices and information security. These devices can completely dissolve upon exposure to water or within a certain period after completing their designated functions, avoiding the risks of secondary surgical removal or information leakage [6,53].

The device structures and fabrication technologies for flexible memristors are rapidly advancing toward higher integration density, multifunctionality, and environmental adaptability. The MIM sandwich structure and its crossbar array design provide a foundational architecture for high-density neuromorphic computing, while unconventional structures expand their application in wearable electronics. Breakthroughs in low-temperature vacuum deposition and solution-based printing technologies have resolved the compatibility challenges between flexible substrates and high-performance functional layers. Systematic mechanical stability evaluation and environmental adaptability studies further lay the groundwork for the practical application of flexible memristors.

## 5. Functions and Applications of Flexible Memristors

Leveraging their nonvolatility, multilevel resistance states, low power consumption, and compatibility with flexible substrates, flexible memristors have become core components for building next-generation flexible electronic systems. Compared with traditional rigid memristors, flexible memristors not only operate stably under mechanical deformation but also integrate information storage, computation, and sensing. Their functions and applications mainly revolve around four major directions: neuromorphic computing, in-memory computing, biomimetic sensing, and biomedical/wearable systems [15,115]. The following sections elaborate on the latest research progress in these four directions.

### 5.1. Neuromorphic Computing

The core of neuromorphic computing lies in emulating the highly parallel, low-power information processing mode of the human brain. Flexible memristors, owing to their continuously tunable conductance, are widely used to emulate biological synaptic plasticity, serving as the foundation for constructing flexible neuromorphic computing hardware.

#### 5.1.1. Synaptic Plasticity Emulation

Biological synaptic rules such as short-term plasticity (STP), long-term plasticity (LTP), and spike-timing-dependent plasticity (STDP) are the cornerstones of learning and memory [116,117,118,119]. Although traditional rigid memristors can emulate these functions, they often suffer from performance degradation under mechanical bending. To address this issue, researchers have developed a series of flexible artificial synapses that operate stably under deformation through interface engineering and material innovation [50,120,121]. Wu et al. [122] introduced an Al_2_O_3_ diffusion barrier layer into a TiO_x_/Al_2_O_3_ bilayer structure, effectively slowing down the migration rate of oxygen vacancies. This allowed the device to stably emulate STDP and PPF even after 1000 bending cycles, and significantly optimized the nonlinearity of LTP/LTD from 2.57/8.2 (single-layer device) to 1.62/1.46. Similarly, Panda et al. [116] introduced HfOx as an ion diffusion limiting layer in a WO_x_/HfO_x_ structure, not only reducing the switching nonlinearity to 0.68/1.98 but also achieving a high transmittance of 92.2%, providing a new approach for constructing high-precision transparent flexible synapses.

#### 5.1.2. Pattern Recognition Accuracy

Using flexible memristor arrays for image recognition is key to verifying their synaptic performance. Researchers utilize device arrays to emulate the potentiation and depression of synaptic weights, thereby performing pattern recognition tasks on standard datasets (e.g., MNIST). Benefiting from improvements in the linearity and symmetry of conductance modulation, flexible memristor arrays have stably achieved recognition accuracies exceeding 90%. However, behind the differences in device accuracy discussed above, there are clear quantitative rules. Wang et al. [11] directly verified the decisive influence of nonlinearity on accuracy using Pt/VO_x_/TiN memristors: with constant-amplitude pulses, the LTP/LTD nonlinearity factors were as high as 4.08/4.17, and the CNN recognition accuracy for pressure–temperature multimodal signals was only 79.16%; after adopting an incremental pulse strategy, the nonlinearity factors improved to 0.69/2.20, and the accuracy of the same network structure jumped to 98.25%, corresponding to about 5.7 percentage points of accuracy improvement per unit reduction in nonlinearity. Lu et al. [123] further revealed the trade-off between ON/OFF ratio and linearity in GaSe vertical memristors: when the HfO_2_ interface layer thickness increased from 1 nm to 3 nm, the ON/OFF ratio increased from 30 to 5.9 × 10^5^, but the device simultaneously degraded from analog to abrupt digital switching, and the LTP/LTD nonlinearity deteriorated sharply, causing the MNIST recognition accuracy to drop from above 93% to its lowest level, demonstrating that simply pursuing a high ON/OFF ratio without regard to linearity optimization cannot translate into system-level benefits; linearity is the more fundamental factor determining the accuracy ceiling. Furthermore, regarding the impact of device variability on system accuracy, Kimura et al. [67] injected Gaussian-distributed random variations into each conductance value of an AOS memristor array during the inference phase, quantitatively evaluating the relationship between variability and recognition accuracy. When the conductance standard deviation increased from 0% to 20%, the MNIST recognition accuracy remained stable at 94.1%, providing a safe tolerance boundary for variability under this system, indicating that within the threshold, array-level integration is almost unaffected by device-to-device differences. These results collectively reveal the mapping rules from device parameters to system accuracy: nonlinearity determines the theoretical upper limit of accuracy; the improvement of ON/OFF ratio must be premised on maintaining linearity; and conductance variation within 20% has basically no effect on accuracy. These quantitative relationships provide measurable design guidelines for moving flexible memristors from single-device optimization to array-level system integration.

Table 4 summarizes the performance metrics of representative works. As shown in Table 4, through material system innovation (e.g., perovskites, MOFs) and interface engineering, flexible memristor arrays have reached levels comparable to ideal software in handwritten digit recognition tasks, laying a solid foundation for wearable neuromorphic computing hardware.

### 5.2. In-Memory Computing: Integration of Storage and Logic

In-memory computing aims to break through the “memory wall” bottleneck caused by the separation of processing units and memory units in the traditional von Neumann architecture. Memristors, with their nonvolatility and multilevel storage capability, have become core elements for implementing in-memory computing.

#### 5.2.1. Multilevel Nonvolatile Storage

**Figure 6:**
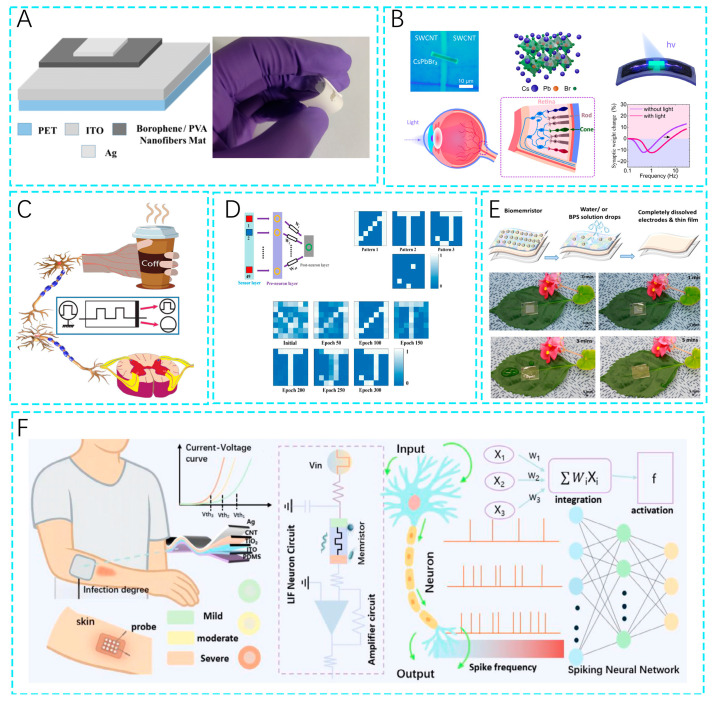
Applications of flexible memristors: (**A**) Structural schematic of a memristor constructed from composite nanofibers [129]. (**B**) Schematic of an intelligent perception system based on flexible memristors [131]. (**C**) Schematic of the working mechanism of a biological nociceptor responding to external harmful stimuli [132]. (**D**) Learning results of the (BZA)_2_CuBr_4_ memristor [133]. (**E**) Schematic of the degradation process of a bio-based memristor over time [53]. (**F**) Principle schematic of a biomimetic neuromorphic wound infection monitoring system [134].

Flexible memristors can achieve multiple discrete and stable resistance states by adjusting the compliance current or pulse parameters, thereby storing multi-bit data in a single device. Das et al. [129] fabricated a borophene-PVA composite nanofiber memristor using electrospinning technology. Leveraging the high charge carrier mobility of borophene, the device achieved an ON/OFF ratio of approximately 4 × 10^3^ and a data retention time exceeding 10^4^ s (Figure 6A). Vu et al. [130] reported a floating-gate memristor based on a large-area MoS_2_/graphene heterojunction. Utilizing a reliable charge trapping mechanism, the device achieved a high ON/OFF ratio exceeding 10^4^, endurance of over 8000 cycles, and stable multilevel storage capability.

#### 5.2.2. Logic Operations and Crossbar Arrays

In addition to data storage, performing logic operations directly within flexible memristors is another key pathway for in-memory computing. Meng et al. [7] constructed flexible memristors based on boron nitride nanosheets and successfully implemented implication (IMP) logic by building two-device-one-resistor (2R1R) and three-device circuits, with energy consumption per synaptic event as low as 198 fJ. To achieve high-density integration, crossbar array structures are widely adopted, but they face the challenge of “sneak path currents.” Kim et al. [135] introduced an ethoxylated polyethyleneimine (PEIE) polymer interfacial layer into a dinaphthothienothiophene (DNTT) organic semiconductor, fabricating flexible memristors with self-rectifying characteristics. The extremely low reverse leakage current effectively suppressed array crosstalk without the need for additional selector devices. Building on the logic operations and array architectures described above, combining spiking neural networks (SNNs) with memristor crossbar arrays has become a key direction for achieving energy-efficient neuromorphic computing. Deepthi et al. [108] designed an adaptive leaky integrate-and-fire (LIF) neuron circuit using volatile and nonvolatile memristors, and constructed 2 × 2 and 5 × 5 SNN crossbar arrays. By eliminating the reset circuit, they significantly reduced the energy per spike and improved scalability. Karamimanesh et al. [76] proposed a fully CMOS-implemented LIF neuron that generates bipolar output spikes to directly modulate memristor synaptic conductance, achieving local STDP learning. In a 15 × 4 SNN, they verified on-chip learning and pattern association capabilities, with training and inference requiring only 0.6 ms and 0.32 ms, respectively. Lee et al. [21] constructed a 32 × 32 crossbar array of self-rectifying charge-trap memristors. By employing a hardware-aware training method to overcome the issues caused by non-ohmic I-V characteristics, they achieved a classification accuracy of 93.5% in real-time electrocardiogram (ECG) diagnosis, with energy consumption reduced by four orders of magnitude compared to a graphics processing unit (GPU).

### 5.3. Biomimetic Sensing: From Sensation to Recognition

Flexible memristors not only excel in computing but also show great potential in emulating biological sensory functions, evolving from simple tactile and pain sensation simulation toward multimodal, closed-loop intelligent perception systems [131] (Figure 6B).

#### 5.3.1. Tactile and Pain Sensation

In the area of tactile and pain sensation, an ITO-based flexible transparent memristor, without the need for electroforming, emulated the threshold, non-adaptation, and relaxation behaviors of nociceptors, successfully distinguishing between harmful and harmless stimuli [136]. From the perspective of application requirements, tactile and pain perception require memristors to possess threshold switching characteristics, volatile resistive switching behavior, and sensitive response to pulse intensity and timing, in order to simulate key functions of biological nociceptors such as “threshold triggering–relaxation–non-adaptation” [132,136]. Based on this, volatile threshold memristors with a diffusive conductive filament mechanism are the optimal choice, because their spontaneous recovery and cumulative excitation characteristics naturally match the dynamic response patterns of biological receptors. Panda et al. [132] further demonstrated an all-oxide ITO/ZnO/TiO_2_ memristor that emulated more complex sensitization phenomena such as hyperalgesia and allodynia (Figure 6C). To achieve closed-loop perception, Huang et al. [137] integrated an AgNW memristor-based spiking neuron with a dual-mode pressure-temperature sensor. The analog signals were converted into spike trains, classified by a convolutional neural network, achieving 100% recognition accuracy across four perception levels and successfully driving a robotic hand to perform avoidance reflexes.

#### 5.3.2. Optoelectronic and Olfactory Sensation

Optoelectronic and olfactory biomimetic sensing place core requirements on memristors for photoelectric/gas-sensitive multimodal synergistic modulation capability and nonvolatile multilevel state retention, to achieve effective mapping of external information such as light intensity/wavelength or gas concentration to synaptic weights [133,138]. Therefore, nonvolatile resistive switching memristors with photo- or gas-induced conductance modulation (such as those based on charge trapping or defect migration mechanisms) are the most ideal hardware platforms for such applications. Liu et al. [138] reported a Cu_3_P nanobelt-based optoelectronic memristor that emulated STDP and PPF under dual modulation of light and electricity, and demonstrated a “learning-forgetting-relearning” pattern recognition process using a 5 × 5 array. Chen et al. [133] developed a lead-free two-dimensional perovskite (BZA)_2_CuBr_4_ memristor, in which the operating voltage was significantly reduced under ultraviolet light illumination. Its 7 × 7 array successfully recognized specific patterns in unsupervised learning (Figure 6D). Furthermore, Gao et al. [139] fabricated a broadband optoelectronic memristor based on an aluminum-doped zinc oxide (AZO) homojunction, which exhibited optical synaptic plasticity in the range of 380–620 nm. Its 20 × 20 array achieved color image perception and storage, and a single-image super-resolution neural network was successfully constructed. In olfactory biomimetics, Lu et al. [140] integrated a NiO gas sensor, a flexible oscillator, and an GO/chitosan memristor into a hybrid system. This system encoded H_2_S concentration into spike frequency and performed perceptual learning using a K-nearest neighbor algorithm, significantly reducing recognition error rates.

### 5.4. Biomedical and Wearable Systems

Flexible neuromorphic memristors exhibit broad application prospects in implantable medical devices, transient electronics, smart textiles, and health monitoring due to their excellent biocompatibility, mechanical flexibility, and low power consumption.

#### 5.4.1. Implantable and Transient Electronics

For implantable and transient electronics, memristors must meet core requirements such as biocompatibility, degradability, long-term stability in physiological environments, and ultra-low power consumption [53]. Considering the advantages and disadvantages of various resistive switching mechanisms, conductive filament (ECM) memristors based on degradable metal electrodes (such as Mg, W) and bio-electrolyte materials are the optimal choice for achieving controllable transient degradation while maintaining stable resistive switching performance. Zhu et al. fabricated flexible memristors based on ZrO_x_ thin films and verified their stable resistive switching behavior (ON/OFF ratio > 10^3^) in ex vivo porcine tissue, providing hardware foundations for human–machine interaction and real-time physiological signal monitoring [141]. Transient and degradable electronics focus on devices that can be dissolved by bodily fluids after completing their tasks. Raeis-Hosseini et al. [53] used fish-scale collagen as the electrolyte and Mg as the electrode to fabricate bio-memristors that completely dissolve within minutes (Figure 6E). The devices maintained stable performance after 1000 bending cycles at a bending radius of 5 mm, and a neural network based on these devices achieved an accuracy of 92.2% in MNIST recognition.

#### 5.4.2. Wearable Electronics and Smart Textiles

Wearable and smart textile systems are one of the most promising application directions for flexible memristors [142]. Their core requirements include excellent mechanical flexibility, low-power operation, and conformal integration compatibility with fabrics. In this scenario, conductive filament (ECM/VCM) and nonvolatile interface-type memristors based on organic/polymer materials or low-dimensional inorganic materials, owing to their intrinsic flexibility and solution processability, are the best candidates for building intelligent textile neuromorphic computing systems. Jiang et al. [103] fabricated textile memristors (ON/OFF ratio > 10^5^) using carbon fibers as electrodes and a BiOI/TiO_2_ heterointerface as the functional layer. Integrated with pressure sensors, they built a smart textile system that achieved 98.5% accuracy in gesture recognition. Guan et al. [104] fabricated textile memristors based on Cu-TCPP porphyrin materials via electrophoretic deposition, achieving an ultra-low reset voltage of −0.037 V and an ultra-high ON/OFF ratio of 5 × 10^7^. A system integrating temperature, light, and pressure sensors could assist visually impaired individuals in recognizing Braille characters. For health monitoring, Gosai et al. [143] fabricated a 3 × 3 Ti_3_C_2_T_x_ MXene crossbar array, which was directly attached to the forearm skin to acquire four-channel electromyography (EMG) signals. The system successfully distinguished ten hand gestures and achieved nearly 100% pattern recognition accuracy by the 9th training epoch. Zhang et al. [134] designed an Ag/CNT-TiO_2_ threshold memristor whose threshold voltage decreased from 2.7 V to 0.8 V with increasing wound infection severity. When integrated with a leaky integrate-and-fire (LIF) neuron, the infection signal was converted into spike frequency, and a spiking neural network achieved a classification accuracy of 93.8% for infection levels (Figure 6F).

Flexible memristors have achieved key breakthroughs in four major directions: neuromorphic computing, in-memory computing, biomimetic sensing, and biomedical/wearable systems. Through material innovation and structural optimization, devices can faithfully reproduce various forms of synaptic plasticity, providing hardware foundations for high-precision pattern recognition. The introduction of self-rectifying crossbar arrays effectively suppresses sneak path currents, significantly enhancing the energy efficiency of in-memory computing. Multimodal biomimetic sensing systems have achieved a leap from single-sensation to closed-loop intelligence. Wearable and implantable applications fully demonstrate their unique advantages in biocompatibility and transient degradation. Although device uniformity and long-term stability remain major challenges, flexible memristors undoubtedly open up new development pathways for next-generation wearable artificial intelligence and edge computing hardware.

## 6. Challenges and Outlook

Flexible memristors have made considerable progress in functional emulation at the single-device level, but the core bottleneck for their practical application is shifting from “whether the function can be realized” to “whether it can work reliably and long-term under dynamic deformation environments, and support system-level intelligent tasks with integrable architectures.” This chapter starts from electrical non-idealities and system integration bottlenecks, and then looks forward to the development path from device optimization to sensing–memory–computing integrated systems.

### 6.1. Electrical Non-Idealities and System Integration Bottlenecks

The transition of flexible memristors from the laboratory to practical arrays faces severe electrical non-idealities. Large device-to-device and cycle-to-cycle variations in resistive switching parameters, significant fluctuations in operating voltages, together with inherent electrical noise and relaxation effects, not only degrade the retention and endurance of individual devices but also cause crosstalk and extra power consumption in crossbar arrays. Shooshtari et al. [23] pointed out in their review that the non-idealities of memristors originate from the intrinsic randomness of conductive filament formation and rupture, stochastic ion migration, trap-state dynamics, and material inhomogeneity. In flexible systems, the stress field introduced by mechanical bending couples with the electric and thermal fields, further exacerbating the uncontrollability of filament evolution. Notably, Ding et al. [144] showed that such noise is not entirely detrimental: as a “perturbator,” it can help combinatorial optimization escape local minima; as a “generator,” it can be used for physical unclonable functions, stochastic computing, and Bayesian neural networks, demonstrating a paradigm shift from combating non-idealities to actively utilizing them.

To address these challenges, researchers are exploring solutions at both the device optimization and system architecture levels. At the device level, Lee et al. [127] systematically compared the effects of α-Ta and β-Ta bottom electrodes on the performance of UVO-oxidized TaO_x_ devices, finding that the electrode crystal phase directly determines the defect density and distribution in the oxide layer. The α-Ta electrode kept cycle-to-cycle variation within 10.14%, while the β-Ta electrode, due to higher oxygen vacancy concentration, caused diffuse switching voltage distribution and cycle-to-cycle variation exceeding 20%. Guan et al. [128] proposed a design–technology co-optimization strategy, obtaining MoS_2_ nanosheets with highly convergent thickness and lateral size through electrochemical intercalation exfoliation. The geometric confinement effect of prefabricated voids formed by nanosheet stacking constrained conductive filament evolution to specific paths, narrowing the distribution widths of V_set_ and V_reset_ to ±0.45 V and ±0.37 V, respectively, with a device yield of 93.75%. At the system level, establishing hardware non-ideality models that incorporate variability and noise and embedding them into training algorithms for fault-tolerant compensation can achieve a substantial improvement in array yield at the cost of a small loss in accuracy (<5%) [145]. Liu et al. [146] designed a coupled dual-channel memristor that achieves orthogonal modulation of the weight mean (μ) and standard deviation (σ) through a differential readout architecture, synthesizing arbitrary Gaussian distributions in hardware for Bayesian neural networks, thus incorporating device noise into the probabilistic computing framework rather than simply suppressing it. These studies indicate that combining device physics innovation with algorithm–hardware co-design can transform non-idealities from constraints into hardware resources that support uncertainty quantification and robust edge computing.

### 6.2. Outlook: From Device Optimization to Sensing-Memory-Computing Integrated Systems

For the future development of flexible neuromorphic memristors, the first step is to overcome the bottlenecks of electrical reliability and mechanical robustness in dynamic bending environments at the device level. Wang et al. [147] constructed long-range-ordered Cu_12_Sb_4_S_13_ quantum dot superlattices, utilizing strong quantum coupling effects to control resistance-state variation within 0.1% over 8.4 × 10^7^ s of continuous operation and 10^6^ fast read cycles, with a nonlinearity factor as low as 2.03, providing a material engineering paradigm for high-reliability flexible neuromorphic computing. At the same time, self-healing structures open new paths for extending device lifetime under repeated deformation. Bhise and Kim [148] systematically reviewed the application of intrinsic self-healing mechanisms based on dynamic covalent bonds, hydrogen bonds, and metal–ligand coordination in organic memristors and transistors, pointing out that embedding self-healing functions in the switching layer can effectively suppress microcrack propagation and maintain synaptic weight stability under repeated bending. These advances indicate that through synergistic atomic-scale ordered assembly and molecular-level dynamic repair, flexible memristors are expected to significantly improve their service life in complex mechanical environments while maintaining low power consumption and synaptic plasticity.

A more profound breakthrough lies in innovation at the system architecture level, namely building a closed-loop “sensing–memory–computing” integrated intelligent system that fuses sensor signal acquisition, spiking neuron encoding, and nonvolatile weight storage on the same flexible platform. Li et al. [102] constructed a 3D crossbar array without interlayer isolation layers by alternately stacking p-type and n-type silicon nanowires, utilizing the rectifying characteristics of the built-in pn junction (rectification ratio up to 10^5^) to fundamentally eliminate sneak currents, providing a feasible hardware path for multilayer flexible neuromorphic integration. Lan et al. [149] systematically reviewed the progress of memristor applications in the biomedical field, pointing out that memristor arrays on flexible substrates have achieved preliminary real-time acquisition and parallel processing of multimodal physiological signals such as pressure, temperature, and humidity, but still lack end-to-end closed-loop demonstrations from perception to decision-making. In the future, it is necessary to establish hardware non-ideality models incorporating device variability and noise and embed them into training algorithms for fault-tolerant compensation, thereby trading a small loss in accuracy for a substantial improvement in array yield. At the same time, priority should be given to developing 3D heterogeneous integration and CMOS-compatible processes, and synergistically introducing new paradigms such as self-healing structures and physical reservoir computing into flexible platforms. Only in this way can flexible memristors truly achieve the leap from a single synaptic unit to a full-system intelligence in dynamic and complex environments, providing a solid technological foundation for emerging fields such as wearable artificial intelligence, intelligent healthcare, and edge computing.

## Figures and Tables

**Table 1:** Performance summary of flexible memristors based on natural and bio-derived materials.

Material System	Switching Mechanism	On/Off Ratio	Application Performance
Silk fibroin/CNT [50]	CNT rearrangement/Trap regulation	10^2^	Triplet-STDP, BCM learning rule, direction selectivity simulation
Wool keratin [51]	ECM/tunneling	10^3^	SET@0.54 V, bending radius 1.2 mm (1000 cycles), STDP/PPF/LTP/LTD, 95.8% recognition accuracy
Egg white [52]	ECM (Fe ions)	5 × 10^2^	PPF/PPD conversion, STP; LTP, dissolvable, transmittance > 90%, bending ~5 mm (1000 cycles)
Collagen [53]	ECM (Mg filaments)	20	SET@1.44 V, bending radius 5 mm (1000 cycles), LTP/LTD, 92.2% MNIST accuracy, fully biodegradable
Starch [54]	ECM (Ag filaments)	2 × 10^7^	SET@2.3 V, spike firing, spatiotemporal integration, gain modulation
Chitosan/SWCNT [55]	ECM (ion adsorption/filaments)	14.98	SET@1.5 V/RESET@ −1.5 V, STDP/PPF/LTP/LTD
Lignin [56]	Carbonized filaments	10	±0.7 V pulses, EPSC, SRDP, STP, LTP

**Table 2:** Performance summary of flexible memristors based on synthetic organic and polymer materials.

Material System	Switching Mechanism	On/Off Ratio	Application Performance
Al/Graphene/Parylene/W [63]	ECM (Al filaments)	10^3^	Reset current < 50 μA, power consumption < 150 μW; flexible and transparent
Ag/PPX-Ag/ITO (Parylene with Ag NPs) [40]	ECM (Ag filaments, nanoparticle-assisted)	10^3^	Second-order effects (thermal modulation); STDP
Ag/ZnO NS@PMMA/Pt (on PI) [64]	Trap regulation (oxygen vacancies)	10^2^	SET/RESET@ ±0.4 V; bending R = 5 mm (1000 cycles); MNIST accuracy 97.7%; STDP/LTP/LTD
ITO/PEDOT:PSS/Pentacene/Al (on PEN) [58]	Heterojunction interface regulation/space charge	10	Low power consumption; bending R = 2.5 mm (1000 cycles); MNIST accuracy 92.6%; PPF/SDDP/SRDP
Ag/Pentacene/Ag (on PET) [65]	Trap regulation (SCLC)	1.5 × 10^3^	Bending R = 1.26 cm (1000 cycles); MNIST accuracy 93.8%; coexistence of digital/analog resistive switching; PPF/LTP/LTD
Au/DPDA/ITO (on PET) [59]	Molecular chain torsion (NDR)	10	Energy consumption ~29.9 fJ; bending R = 5.9 mm; MNIST accuracy 95.7%; PPF/BCM/edge detection
ITO/PVA-GO/ITO (on PET) [66]	Valence change (GO reduction/oxidation)	10^3^	SET/RESET@ < 0.2 V, SET power consumption ~0.5 μW; bending R = 5 mm; Pavlovian conditioning
Cu/CuS-PEO/ITO [67]	ECM (Cu ions, CuS nanoparticles)	10^4^	Analog nociceptor simulation (threshold/non-adaptation/relaxation/allodynia/hyperalgesia)
s-SWCNTs/PEO:LiClO_4_ (on PI) [68]	Trap filling (protons)	/	Bending R~6 mm; simulation of pain perception and nerve injury (neuropraxia/axonotmesis/neurotmesis)

**Table 3:** Performance summary of flexible memristors based on inorganic functional materials and nanofillers.

Material System	Switching Mechanism	On/Off Ratio	Application Performance
TaO_x_/HfO_x_ bilayer [30]	Oxygen vacancy concentration regulation	10^2^	SET/RESET@ ±1 V; bending radius 4 mm (>1000 cycles); STDP; LTP/LTD; MNIST accuracy > 96%
ZnO [73]	Trap regulation (SCLC, oxygen vacancies)	10^2^	SET/RESET@ ±0.7 V; bending radius 5 mm; STDP; PPF/PPD; STP; LTP; MNIST accuracy 97.7%
VO_2_ [42]	Thermally induced IMT	/	Driving current < 130 µA; bending radius 15 mm (>1000 cycles); oscillation frequency 16.8 kHz@20 °C (60.2 kHz@40 °C)
CeO_2_ (Ag/ITO) [74]	ECM (Ag filaments) + VCM (oxygen vacancies)	10^7^	Bending radius 15 mm (80 cycles); can simulate neurons (threshold/integration) and synapses (PPF/STP/LTP); volatile/non-volatile dual-mode switching
α-GTO [75]	Conductive component (Vo or H^+^ gradient distribution)	448	SET@ ~4 V; excellent analog characteristics (large dynamic range)
IGZO:H [76]	Trap regulation (H-induced sub-bandgap states)	179 (UV)/93 (Blue)/12 (Green)	4 µm^2^ miniaturization; sensitive to visible light (green/blue); energy consumption 0.01 fJ/pulse (blue/green light); STP; LTM; PPF; STDP
AOS (a-IGZO TFT, a-GTO) [77]	Conductive component gradient	/	Frequency ~33 kHz (projected up to 660 kHz); system MNIST accuracy 94.1%
Laser-induced graphene (LIG) [78]	Built-in electric field	20	Switching@ ±3 V; flexible; short-term plasticity (PPD); can build LIF neuron circuits
GO/Pyridinium salt/GO [36]	Interface regulation	/	Short pulses; STP; LTP; PPF; STDP; SRDP; learning-forgetting-relearning
BP/HfO_x_ bilayer [79]	ECM (Cufilaments)	10^2^	LTP/LTD (1000 pulses); STM, LTM (light intensity/time modulation); learning-forgetting-relearning; 6 × 6 array for visual perception simulation; bending radius 10 mm
(4-FBA)_2_PbBr_4_ [80]	ECM (Ag filaments) + VCM (Br^−^/VBr)	10^3^	Bending radius 10 mm; SET@0.4 V; endurance 340 cycles; retention 10^4^ s; PPF/PPD; LTP/LTD; analog synaptic plasticity simulation

**Table 4:** Performance comparison of representative flexible memristors in neuromorphic computing.

Material System	Device Structure	Synaptic Plasticity	Nonlinearity (LTP/LTD)	Recognition Accuracy	Mechanical Stability
TiO_x_/Al_2_O_3_ [122]	Pt/TiO_x_/Al_2_O_3_/Pt/ITO	PPF, STDP	1.62/1.46	91.15% (MNIST)	1000 cycles
WO_x_/HfO_x_ [116]	ITO/WO_x_/HfO_x_/ITO/PEN	STDP	0.68/1.98	100% (Hopfield)	500 cycles
Co-TCPP nanosheets [124]	Ag/Co-TCPP @PVP/Pt	EPSC, PPF, LTP/LTD	0.48/0.37	95.99% (MNIST)	2000 cycles
CsPbI_3_ perovskite [125]	ITO/PEDOT:PSS/CsPbI_3_/PMMA/Ag	PPF, STDP	/	97.31% (MNIST)	/
ZrO_2_/ZnO heterojunction [126]	Au/ZrO_2_/ZnO/ITO/PET	STP, PPF, STDP	2.28/2.21	97.9% (MNIST)	/
AOS (a-IGZO, a-GTO) [77]	AOS TFT-based neuron/AOS GTO memristor/Schottky diode synapse	/	/	94.1% (MNIST)	/
VO_x_ [127]	Pt/VO_x_/TiN	PPF, STDP, SRDP, SADP, LTP/LTD	0.69/2.20	98.25% (multimodal signals)	/
GaSe/HfO_2_ [128]	Cu/GaSe/Hf O_2_/Au	PPF, PPD, LTP/LTD	/	93% (MNIST)	/

## Data Availability

No new data were created or analyzed in this study. Data sharing is not applicable to this article.
